# Utility of Population-Level DNA Sequence Data in the Diagnosis of Hereditary Endocrine Disease

**DOI:** 10.1210/js.2017-00330

**Published:** 2017-11-15

**Authors:** Paul J. Newey, Jonathan N. Berg, Kaixin Zhou, Colin N.A. Palmer, Rajesh V. Thakker

**Affiliations:** 1 Division of Molecular & Clinical Medicine, Ninewells Hospital & Medical School, University of Dundee, Dundee, DD1 9SY, Scotland, United Kingdom; 2 Academic Endocrine Unit, Radcliffe Department of Medicine, University of Oxford, Oxford, OX3 7LJ United Kingdom; 3 Clinical Genetics, Ninewells Hospital & Medical School, University of Dundee, Dundee, DD1 9SY Scotland, United Kingdom

**Keywords:** ExAC, genetic testing, germline, mutation, penetrance, single nucleotide variant

## Abstract

**Context:**

Genetic testing is increasingly used for clinical diagnosis, although variant interpretation presents a major challenge because of high background rates of rare coding-region variation, which may contribute to inaccurate estimates of variant pathogenicity and disease penetrance.

**Objective:**

To use the Exome Aggregation Consortium (ExAC) data set to determine the background population frequencies of rare germline coding-region variants in genes associated with hereditary endocrine disease and to evaluate the clinical utility of these data.

**Design, Setting, Participants:**

Cumulative frequencies of rare nonsynonymous single-nucleotide variants were established for 38 endocrine disease genes in 60,706 unrelated control individuals. The utility of gene-level and variant-level metrics of tolerability was assessed, and the pathogenicity and penetrance of germline variants previously associated with endocrine disease evaluated.

**Results:**

The frequency of rare coding-region variants differed markedly between genes and was correlated with the degree of evolutionary conservation. Genes associated with dominant monogenic endocrine disorders typically harbored fewer rare missense and/or loss-of-function variants than expected. *In silico* variant prediction tools demonstrated low clinical specificity. The frequency of several endocrine disease‒associated variants in the ExAC cohort far exceeded estimates of disease prevalence, indicating either misclassification or overestimation of disease penetrance. Finally, we illustrate how rare variant frequencies may be used to anticipate expected rates of background rare variation when performing disease-targeted genetic testing.

**Conclusions:**

Quantifying the frequency and spectrum of rare variation using population-level sequence data facilitates improved estimates of variant pathogenicity and penetrance and should be incorporated into the clinical decision-making algorithm when undertaking genetic testing.

## Teaser

The frequency of germline coding-region variation was evaluated in 38 endocrine disease genes in 60,706 individuals. The clinical utility of these data in improving genetic diagnosis is demonstrated.

The advent of high-throughput DNA sequencing methods has accelerated the identification of genes responsible for hereditary disorders, and they are increasingly applied in clinical practice to guide patient management [1–3]. The potential utility of these approaches are heralded by ambitious clinical projects, including the United Kingdom’s 100,000 Genomes Project and the United States‒based Regeneron/Geisinger DiscovEHR collaboration, which are combining next-generation sequencing with health care data to facilitate gene discovery and precision medicine [4, 5]. However, this accessibility and scope of germline genetic testing brings many challenges, including inherent difficulties in data interpretation [6, 7]. For example, failure to identify pathogenic variants in an individual may result in missed opportunities for diagnosis and treatment, whereas misclassification of benign variants as pathogenic may result in inadvertent harm through unnecessary investigation and treatment [7]. Thus, appropriate genetic counseling and treatment of patients and their families relies on accurate estimates of the pathogenicity of genetic variants and their clinical penetrance. However, it is important to note that frequently these have been overestimated because of focusing genetic analysis on disease cohorts without adequate investigation of control populations; an overreliance on *in silico* computational tools in predicting variant effects; and self-fulfilling reporting or ascertainment bias in the literature [8–10].

The detailed genetic characterization of large population cohorts provides an unbiased resource to reevaluate the role of germline genetic variation in hereditary disease, as illustrated by results from the exome variant server and 1000 Genomes Project cohorts, which identified a surprising high degree of rare coding-region variation as well as demonstrating that many disease-associated variants reported as pathogenic were instead observed with improbably high frequencies in apparently healthy individuals, indicating likely misclassification [11–13].

The Exome Aggregation Consortium (ExAC) data set, containing high-resolution exome sequences from 60,706 unrelated individuals [14], provides the most comprehensive publicly available catalog of population-level coding-region variation and confirms this remarkable diversity in rare coding-region variation [*i.e.,* >99% of all single-nucleotide variants (SNVs) occur with an allele frequency (AF) of <1%] and that every individual will harbor a large number of apparently deleterious/pathogenic alleles with imperceptible impacts on health [14]. The potential utility of this data set to reassess variant pathogenicity and to refine estimates of disease penetrance (*i.e.,* the proportion of individuals with a particular variant-expressing disease) has recently been demonstrated for hereditary forms of prion disease and cardiomyopathy [8, 9, 15]. Indeed, evaluating the frequency of variants identified from clinical genetic testing in large control populations, such as ExAC, is now mandatory, enabling the exclusion of variants occurring with AFs above certain thresholds (*e.g.,* AF >0.1% for dominant disorders) [6, 7]. In contrast, the absence (or very low AF) of a variant from a population database is often used as supporting evidence of pathogenicity [6]. However, the background frequency of rare coding-region variation in the gene of interest will potentially influence the interpretation of the result, although to date, the burden of such rare variation is typically not considered during variant interpretation.

Germline genetic testing is increasingly used in the field of endocrinology, reflecting several recent advances in disease-gene discovery [2, 16]. Indications for genetic testing include the evaluation of individuals at risk for monogenic disease [*e.g.,* multiple endocrine neoplasia type 1 (MEN1)] [17]; sporadic clinical presentations associated with a high prevalence of germline mutations [*e.g.,* pheochromocytoma/paraganglioma (PPGL)] [16]; or investigative studies for clinical presentations in which a genetic etiology is suspected [2]. Increasingly, genetic testing employs next-generation sequencing approaches, including the use of disease-targeted gene panels, and it is inevitable that as the genomic content of the test increases, so does the likelihood of detecting rare coding-region variants, resulting in the potential for diagnostic uncertainty. Indeed, a failure to account for the background frequency of rare variants may have contributed to ascertainment bias in earlier genetic studies, resulting in potential variant misclassification as well as inadvertent overestimates of disease penetrance.

Therefore, to address these challenges, we used the ExAC cohort to quantify the spectrum and frequency of rare germline missense and loss-of-function (LOF) SNVs in 38 genes associated with hereditary endocrine disease and explored the utility of these data when applied to several clinical settings. Our results demonstrate the value of large control cohorts such as ExAC and illustrate how estimates of cumulative rare variant frequencies, together with additional gene- and variant-level factors, may be incorporated into the workflow for clinical genetic testing.

## 1. Materials and Methods

### A. ExAC Population, Genes, and Variant Classification

Data were obtained from the ExAC browser (Version 0.3.1; http://exac.broadinstitute.org; accessed March 2016 to October 2017). Details of the contributing populations, sequencing methods, and variant filtering and calibration methods have been reported [14]. All high-quality nonsynonymous SNVs were identified in the 38 genes selected for study, including those predicted to result in missense or nonsense amino acid changes and those directly disrupting donor or acceptor splice sites. SNVs are described relative to the canonical transcript. This analysis was performed on the complete ExAC data set (n = 60,706). However, a separate subanalysis was performed on the data set with the 7601 germline samples from The Cancer Genome Atlas (TCGA) cohort removed (further details provided in Supplemental Table 1 and the Supplemental Materials and Methods). Similarly, although insertions and deletions (indels) were excluded from the main analysis because of the reduced reliability of detection and an increased false discovery rate relative to SNVs [14], a separate analysis evaluated the frequency of LOF indels (*i.e.,* resulting in a frameshift) in each of the 38 genes (further details are provided in the Supplemental Materials and Methods).

Rare SNVs were further categorized into three nonexclusive groups: those with an AF <0.5% (*i.e.,* affecting <1 in 100 individuals); those with an AF <0.05% (*i.e.,* affecting <1 in 1000 individuals); and singletons (*i.e.,* variants observed only once in the ExAC cohort). SNVs were excluded if the AF exceeded the category cutoff in any of the defined ethnic subpopulations or if <10,000 alleles were captured. Cumulative frequencies of each category of rare variant (*i.e.*, AF <0.5%, AF <0.05%, or singleton) were estimated for each gene by accruing individual SNV frequencies to establish population-level carriage rates and were subsequently converted to a “number needed to sequence,” representing the mean number of individuals sequenced for each rare SNV identified. A separate subanalysis of only LOF SNVs was performed (*i.e.,* single-nucleotide substitution predicting either a nonsense amino acid change or directly affecting a donor or acceptor splice site).

The relationship between the gene-specific cumulative rare variant frequencies and amino acid length of the encoded protein was evaluated by linear regression. Cumulative variant frequencies were subsequently corrected for coding-region nucleotide length and normalized to an arbitrarily selected gene *cell division cycle 73* (*CDC73*). To investigate the influence of evolutionary conservation, pairwise amino acid alignment scores were established for known orthologs (http://www.ncbi.nlm.nih.gov/homologene) (Supplemental Table 2). Genes were ranked in increasing order of size-corrected cumulative SNV frequency and in decreasing order of evolutionary conservation as defined by the degree of amino acid conservation between human and zebrafish (*Danio rerio*) orthologs, selected to represent an evolutionary distant species for which a near-complete data set could be generated, and were evaluated by Spearman rank correlation.

### B. Constraint Metrics and Computational Tools of Variant Pathogenicity

Missense (*z*-score) and LOF [probability of LOF intolerance (pLI)] constraint metrics were obtained directly from the ExAC browser (Supplemental Table 3). Detailed descriptions of these metrics are reported elsewhere, and a brief overview is provided in the Supplemental Appendix [14]. SIFT, Polyphen2, and Combined Annotation Dependent Depletion (CADD) scores were evaluated for all missense SNVs with an AF <0.5% in a subset of 12 genes (downloaded from http://cadd.gs.washington.edu/) [18]. Additional details of these tools are provided in the Supplemental Appendix. Variants were categorized as deleterious if they met all of the following criteria: AF <0.5%; SIFT score ≤0.05; a Polyphen2 description of probably damaging; and a scaled CADD score >20. Variants were considered possibly deleterious when they had an AF <0.5% and either a SIFT score ≤0.05 and/or a Polyphen2 classification of probably damaging or possibly damaging.

### C. Prevalence of Disease-Associated Variants in the ExAC Cohort

The presence of SNVs previously reported to be disease causing for six penetrant monogenic conditions [familial hypocalciuric hypercalcemia (FHH), MEN1, multiple endocrine neoplasia type 2 (MEN2), hyperthyroidism-jaw tumor syndrome, neurofibromatosis type 1 (NF1), and von Hippel-Lindau (VHL) syndrome] were evaluated in the ExAC cohort. Disease-associated mutations were identified from publicly available sources (details are provided in Supplemental Appendix). In a separate analysis, the ExAC data set was screened for actionable variants in *MEN1*, *RET*, *VHL*, *SDHB*, *SDHC*, *SDHD*, and *SDHAF2* (as recommended by the American College of Medical Genetics and Genomics (ACMG) guidelines) [19]. For each analysis, it was established whether variants had arisen in samples from the TCGA cohort.

### D. Prevalence of Missense Aryl Hydrocarbon Interacting Protein (*AIP*) SNVs in the ExAC and Sporadic Pituitary Tumor Cohorts

Individual and cumulative frequencies of all missense *AIP* SNVs with an AF <0.5% were established for the ExAC cohort and compared with those observed in 1866 individuals with apparently sporadic pituitary tumors reported in nine earlier studies. Odds ratios with 95% confidence intervals were established.

### E. Prediction of Background Rare Variant Frequencies in Clinically Relevant Gene Panels

Four disease-targeted gene panels were formulated to model estimated background frequencies of rare missense and LOF SNVs when undertaking multiple-gene sequencing. These represented PPGL, calcium-/parathyroid-related disorders, pituitary tumor disorders, and MEN syndromes. Cumulative rare variant frequencies were used to establish the likelihood that a given individual would harbor a rare variant in at least one of the panel genes.

## 2. Results

### A. Rare Variant Frequencies and Evolutionary Conservation

**Figure 1.**
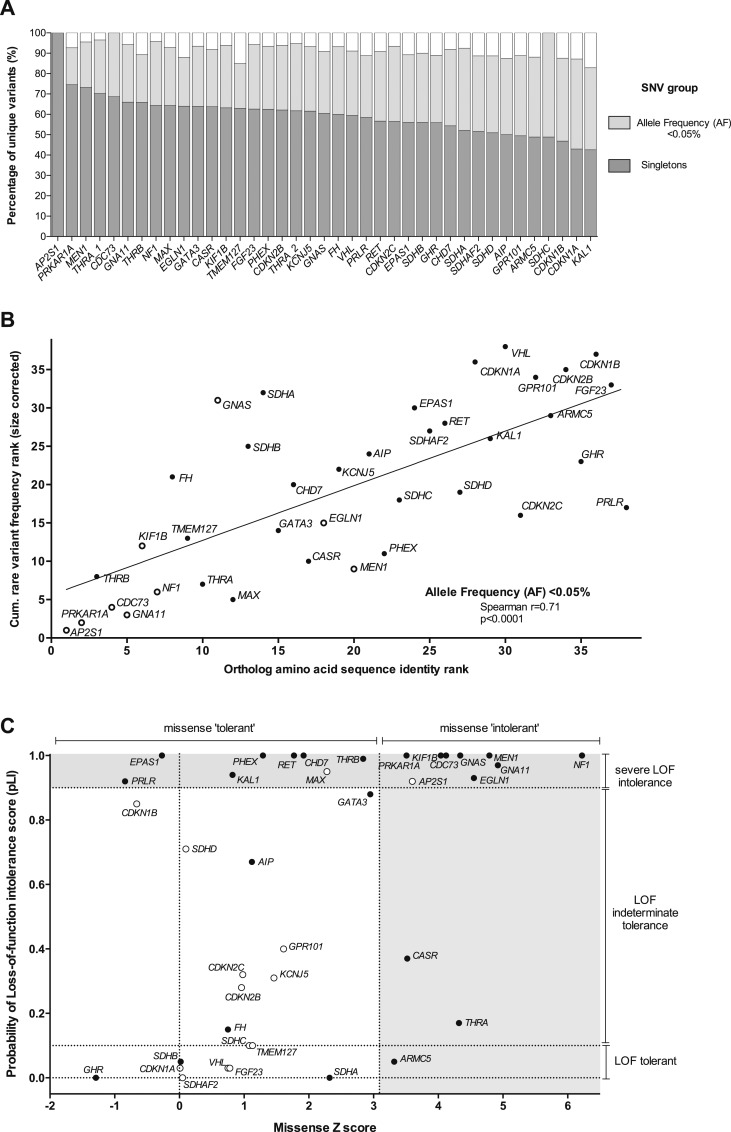
Rare variant frequency, evolutionary conservation, and constraint metrics of genes associated with hereditary endocrine disease. (A) Proportion of all nonsynonymous SNVs occurring in the selected genes as singletons or with an AF <0.05%. Across the 38 genes, an average of 59.8% (range, 42.6% to 100%) of individual missense/LOF SNVs occurred as singletons, whereas 91.8% (range, 83% to 100%) had an AF <0.05%. (B) Rare SNV frequency was correlated with evolutionary conservation of the encoded protein. Individual genes were ranked according to both their size-corrected cumulative SNV frequency (for SNVs with AF <0.05%) and the degree of amino acid conservation between human and zebrafish (*Danio rerio*) orthologs. A significant correlation was observed (*r* = 0.69; *P* < 0.0001), such that genes with a high degree of conservation harbored the lowest rates of rare SNVs. Of note, a marked overlap was observed between genes with high conservation/low variation and those categorized as intolerant of both missense/LOF variation using constraint metrics (genes marked with open circles). All other genes are represented by closed circles. (C) Missense and LOF constraint metrics for the study genes. A *z* score >3.09 is reported to represent significant missense intolerance, whereas a pLI score >0.9 is indicative of extreme LOF intolerance and suggestive of a haploinsufficient function (*i.e.,* gene intolerant to heterozygous LOF). Of note, 45% (17 of 38) of study genes could be classified as extreme LOF intolerant, whereas 32% (12 of 38) were missense intolerant. Several genes in which both missense and LOF mutations were responsible for penetrant monogenic disorders (*e.g.,* *MEN1*, *CDC73*, and *NF1*) clustered in the combined LOF/missense intolerant group. In contrast, several genes in which the role of heterozygous germline variation in disease pathogenesis was less well defined were categorized as LOF (pLI score <0.1) and/or missense tolerant (*e.g.,* *CDKN1A*, *SDHA*, and *GPR101*). However, the reliability of the pLI constraint metric was reduced for genes of small size where few LOF variants were predicted (*e.g.,* <10), and these genes are identified by an open circle (*e.g.,* *CDKN1B, VHL*). All other genes are represented by closed circles. pLI, probability of LOF intolerance.

Thirty-eight genes were selected for study, representing a range of endocrine disorders reported to be associated with heterozygous germline missense and/or LOF SNVs (Table 1). The identification of all nonsynonymous SNVs (*i.e.,* single-nucleotide substitutions resulting in missense or nonsense amino acid changes or directly affecting donor or acceptor splice sites) in each of the 38 genes revealed that the overwhelming majority were rare, with ~60% occurring as singletons (*i.e.,* observed only once in the ExAC cohort), whereas ~92% of gene-specific SNVs had an AF <0.05% (*i.e.,* observed in ≤1 in 1000 individuals) (Fig. 1A). The cumulative frequency of rare nonsynonymous SNVs differed markedly between genes (Table 2). For example, *chromodomain helicase DNA-binding protein 7* (*CHD7*) and *neurofibromin* (*NF1*) demonstrated the highest frequencies of singleton variants (affecting approximately one in 100 and approximately one in 200 of the cohort, respectively), whereas the lowest frequency was observed for *adaptor-related protein complex 2 sigma 1 subunit* (*AP2S1*) (affecting approximately one in 12,000 individuals). Of note, removal of the TCGA subgroup (n = 7601) from the analysis had a minimal impact on cumulative rare SNV frequencies of the genes evaluated (*i.e.,* those associated with hereditary endocrine tumor syndromes), with the exception of *von Hippel-Lindau* (*VHL)*, for which reduced sequence coverage of part of the gene reduced the reliability of the estimates (Supplemental Table 4).

**Table 1.** **Details of the 38 Genes Studied and Their Associated Endocrine Disorders**

**Gene**	**Disorder(s) Associated With Germline Mutation**	**Mutation Type**	**Inheritance Pattern**	**Canonical Transcript**
*AIP*	Familial isolated pituitary adenoma	Het (LOF, MS)	AD (RP)	ENST00000279146
	Sporadic pituitary adenomas	Het (LOF, MS)	Sporadic	
*AP2S1*	Familial hypocalciuric hypercalcemia	Het (MS)	AD	ENST00000263270
*ARMC5*	Familial macronodular adrenal hyperplasia	Het (MS)	AD (RP)	ENST00000268314
	Sporadic macronodular adrenal hyperplasia	Het (MS)	Sporadic	
*CASR*	Familial hypocalciuric hypercalcemia	Het (MS)	AD	ENST00000498619
	Familial isolated hyperparathyroidism	Het (MS)	AD	
	Autosomal dominant hypocalcemia	Het (MS)	AD	
*CDC73*	Hyperparathyroidism-jaw tumor syndrome	Het (LOF, MS)	AD	ENST00000367435
	Sporadic parathyroid carcinoma	Het (LOF, MS)	Sporadic	
*CDKN1A**^a^*	Sporadic parathyroid adenoma	Het (MS)	Sporadic	ENST00000405375
*CDKN1B*	Multiple endocrine neoplasia type 4	Het (LOF, MS)	AD	ENST00000228872
	Sporadic parathyroid and pituitary adenoma	Het (MS)	Sporadic	
*CDKN2B**^a^*	Sporadic parathyroid adenoma	Het (MS)	Sporadic	ENST00000276925
*CDKN2C**^a^*	Sporadic parathyroid adenoma	Het (MS)	Sporadic	ENST00000262662
*CHD7*	Hypogonadotropic hypogonadism type 5/CHARGE	Het (LOF, MS)	AD (RP)	ENST00000423902
*EGLN1*	Sporadic pheochromocytoma/paraganglioma	Het (MS)	Sporadic	ENST00000366641
*EPAS1*	Sporadic pheochromocytoma/paraganglioma	Het (MS)	Sporadic	ENST00000263734
*FGF23*	Autosomal dominant hypophosphatemic rickets	Het (MS)	AD	ENST00000237837
*FH*	Hereditary leiomyomatosis/renal cell carcinoma	Het (LOF, MS)	AD	ENST00000366560
	Sporadic pheochromocytoma/paraganglioma	Het (MS)	Sporadic	
*GATA3*	Hypoparathyroidism/deafness/renal dysplasia	Het (LOF, MS)	AD	ENST00000379328
*GHR*	Laron dwarfism	Homo (LOF, MS)	AR	ENST00000230882
	Idiopathic short stature	Het*^b^* (LOF, MS)	AD (?)	
*GNA11*	Familial hypocalciuric hypercalcemia	Het (MS)	AD	ENST00000078429
	Autosomal dominant hypocalcemia	Het (MS)	AD	
*GNAS*	Pseudohypoparathyroidism type 1a	Het (LOF, MS)	AD*^c^*	ENST00000371100
*GPR101**^a^*	Sporadic acromegaly	Het/Hemi (MS)	Sporadic	ENST00000298110
*KAL1*	Hypogonadotropic hypogonadism type 1	Hemi (LOF, MS)	XLD	ENST00000262648
*KCNJ5*	Familial hypertension	Het (MS)	AD	ENST00000529694
*KIF1B*	Familial pheochromocytoma/paraganglioma	Het (MS)	AD	ENST00000263934
	Sporadic pheochromocytoma/paraganglioma	Het (MS)	Sporadic	
*MAX*	Familial pheochromocytoma/paraganglioma	Het (LOF, MS)	AD (RP)	ENST00000358664
	Sporadic pheochromocytoma/paraganglioma	Het (LOF, MS)	Sporadic	
*MEN1*	Multiple endocrine neoplasia type 1	Het (LOF, MS)	AD	ENST00000337652
*NF1*	Neurofibromatosis	Het (LOF, MS)	AD	ENST00000358273
*PHEX*	X-linked hypophosphatemic rickets	Het/Hemi (LOF, MS)	XLD	ENST00000379374
*PRKAR1A*	Carney complex	Het (LOF, MS)	AD	ENST00000589228
*PRLR*	Hereditary hyperprolactinemia	Het (MS)	AD	ENST00000382002
*RET*	Multiple endocrine neoplasia type 2	Het (MS)	AD	ENST00000355710
	Familial medullary thyroid cancer	Het (MS)	AD	
*SDHA*	Familial pheochromocytoma/paraganglioma	Het (LOF, MS)	AD (RP)	ENST00000264932
	Sporadic pheochromocytoma/paraganglioma	Het (LOF, MS)	Sporadic	
*SDHAF2*	Familial pheochromocytoma/paraganglioma	Het (MS)	AD (RP)	ENST00000301761
	Sporadic pheochromocytoma/paraganglioma	Het (LOF)	Sporadic	
*SDHB*	Familial pheochromocytoma/paraganglioma	Het (LOF, MS)	AD (RP)	ENST00000375499
	Sporadic pheochromocytoma/paraganglioma	Het (LOF, MS)	Sporadic	
*SDHC*	Sporadic pheochromocytoma/paraganglioma	Het (LOF, MS)	Sporadic	ENST00000367975
*SDHD*	Familial pheochromocytoma/paraganglioma	Het (LOF, MS)	AD (RP)*^c^*	ENST00000375549
	Sporadic pheochromocytoma/paraganglioma	Het (LOF, MS)	Sporadic	
*THRA_1**^d^*	Thyroid hormone resistance	Het (LOF,*^d^* MS)	AD	ENST00000450525
*THRA_2**^d^*	Thyroid hormone resistance	Het (MS)	AD	ENST00000264637
*THRB*	Thyroid hormone resistance	Het (LOF, MS)	AD	ENST00000396671
*TMEM127*	Familial pheochromocytoma/paraganglioma	Het (LOF, MS)	AD (RP)	ENST00000258439
	Sporadic pheochromocytoma/paraganglioma	Het (LOF, MS)	Sporadic	
*VHL*	Von Hippel-Lindau (VHL)	Het (LOF, MS)	AD	ENST00000256474
	Sporadic pheochromocytoma/paraganglioma	Het (LOF, MS)	Sporadic	

Abbreviations: AD, autosomal dominant; AR, autosomal recessive; Hemi, hemizygous; Het, heterozygous; MS, missense; RP, reduced penetrance; XLD, X-linked dominant.

^a^ Genes reported to be associated with endocrine disease, although evidence supporting pathogenicity may be limited.

^b^ Evidence supporting heterozygous *GHR* mutations in idiopathic short stature remains unclear.

^c^ Diseases associated with genomic imprinting.

^d^ THRA_1 refers to THRA isoform 1 encoded by the noncanonical transcript ENST00000450525. Disease-associated nonsense and missense mutations in final exon of isoform 1 are reported. THRA_2 refers to THRA isoform 2 encoded by the canonical transcript (ENST00000450525), in which a missense mutation, also present in isoform 1, has been reported.

**Table 2.** **Gene-Level Estimates of Cumulative Rare Nonsynonymous SNV Carrier Frequencies in the Control Cohort**

**Gene**	**Protein Size (AAs)**	**SNV Group:** **AF <0.5%**		**SNV Group:** **AF <0.05%**		**SNV Group:** **Singleton**	
		**Population Frequency** **(%)**	**Number Needed to Sequence (n)**	**Population Frequency** **(%)**	**Number Needed to Sequence (n)**	**Population Frequency (%)**	**Number Needed to Sequence (n)**
*CHD7*	2997	5.2	19	3.0	34	0.9	115
*NF1*	2839	2.1	47	1.5	67	0.5	187
*GNAS*	1037	2.6	38	1.4	71	0.5	208
*KIF1B*	1770	2.0	51	1.2	81	0.4	226
*RET*	1114	3.0	34	1.4	71	0.4	231
*ARMC5*	935	2.8	35	1.2	82	0.3	289
*EPAS1*	870	2.1	47	1.1	87	0.3	310
*CASR*	1078	1.5	67	0.7	149	0.3	354
*FH*	510	0.9	117	0.5	191	0.3	392
*VHL**^a^*	213	0.7	138	0.5	206	0.2	429
*GHR*	638	1.7	58	0.7	141	0.2	474
*PRLR*	622	1.6	61	0.5	190	0.2	504
*PHEX**^b^*	749	0.8	132	0.5	199	0.2	514
*SDHA*	664	1.3	78	0.9	110	0.2	516
*KAL1**^b^*	680	2.4	42	0.8	131	0.2	536
*EGLN1**^a^*	426	0.6	180	0.3	294	0.2	545
*GATA3*	444	0.5	181	0.3	286	0.2	644
*MEN1*	615	0.4	254	0.4	270	0.1	658
*KCNJ5*	419	0.5	195	0.4	228	0.1	734
*GPR101**^b^*	508	2.0	50	0.7	138	0.1	809
*FGF23*	251	0.5	213	0.3	288	0.1	891
*AIP*	330	1.4	70	0.4	272	0.1	923
*THRB*	461	0.6	156	0.3	379	0.1	963
*THRA_2**^c^*	490	0.3	283	0.3	367	0.1	1034
*SDHB*	280	0.6	168	0.4	319	0.1	1048
*CDKN1B*	198	0.9	109	0.4	264	0.1	1124
*CDC73*	531	0.2	468	0.2	468	0.1	1208
*TMEM127**^a^*	238	0.4	258	0.2	544	0.1	1217
*CDKN2B*	138	0.6	161	0.2	439	0.1	1336
*THRA_1*^c^	410	0.2	517	0.1	679	0.1	1509
*PRKAR1A*	381	0.2	432	0.1	828	0.1	1514
*GNA11*	359	0.1	691	0.1	802	0.1	1594
*CDKN1A*	164	1.4	69	0.3	358	0.1	1598
*SDHAF2*	166	0.4	227	0.2	502	0.05	1890
*SDHD*	159	0.2	433	0.1	659	0.04	2245
*CDKN2C*	168	0.2	593	0.1	728	0.04	2302
*SDHC*	169	0.2	625	0.2	625	0.04	2757
*MAX*	160	0.1	837	0.1	1380	0.03	3318
*AP2S1*	142	0.01	12,066	0.01	12,066	0.01	12,066

The number needed to sequence (NNS) equates to the mean number of individuals (reported to the nearest whole number) requiring sequencing to identify a rare variant of each type (*i.e.*, AF <0.5%, AF <0.05%, or singleton). This was determined by taking the reciprocal of the estimated cumulative variant frequency for each category of rare variant per individual (*i.e*., taking into account the presence of two alleles per gene). Genes are arranged in decreasing frequency of singleton variants.

Abbreviation: AA, amino acid.

^a^ Missing data for part of gene and/or reduced reliability of estimates due to reduced exon coverage.

^b^ For X-linked disorders, the NNS is stated for females (*i.e*., accounting for each allele).

^c^ Two transcripts are reported for *THRA* as described in the footnote in Table 1.

Although a positive correlation was observed between rare nonsynonymous SNV frequency and coding-region nucleotide length (*e.g.,* for singleton SNVs*, r^2^*= 0.84; *P* < 0.0001) (Supplemental Fig. 1), marked differences persisted after correcting for gene size (Supplemental Fig. 2). Furthermore, a significant correlation was observed between rare nonsynonymous SNV frequency and the degree of amino acid sequence identity between orthologs of the encoded protein, with the most highly conserved genes (*e.g.,* *AP2S1*, *CDC73*) demonstrating the lowest rates of rare variation (Fig. 1B; Supplemental Table 2).

### B. Hereditary Endocrine Genes Demonstrated Reduced Tolerance of Rare Variation

To further investigate gene-level differences in rare variant frequency, we evaluated the utility of recently reported metrics of constraint, which aim to quantify the deviation between observed and expected numbers of rare nonsynonymous SNVs resulting in either a missense amino acid change or an LOF (*i.e.,* nonsense or donor/acceptor splice site change) (Supplemental Table 3). Of the 38 genes, 20 (53%) were categorized as missense and/or LOF intolerant (Fig. 1C), including the majority of genes associated with monogenic autosomal dominant (*e.g.,* *MEN1, NF1*) or X-linked‒dominant disorders [*phosphate regulating endopeptidase homolog, X-linked* (*PHEX*)], thereby indicating appropriate constraint against nonsynonymous heterozygous variation. In contrast, many disease-associated genes were categorized as missense and/or LOF tolerant, including several in which the role of heterozygous variation and endocrine disease is less established [*e.g.,* *growth hormone receptor* (*GHR*)] or was previously associated with reduced disease penetrance [*e.g.,* *succinate dehydrogenase A* (*SDHA)*, *succinate dehydrogenase B* (*SDHB*)]. For other genes in the tolerant groups [*e.g.,* *cyclin-dependent kinase inhibitor 1B* (*CDKN1B*)], their small size reduced the reliability and utility of the respective constraint metrics (Fig. 1C).

To further quantify the variability in LOF SNV frequency (*i.e.,* resulting in a nonsense amino acid change or disrupting donor/acceptor splice sites), the cumulative estimates of LOF SNV allele frequencies were established for each gene (Supplemental Table 5). Although many genes displayed an absence or very low number of LOF SNV alleles consistent with their known haploinsufficiency function (*e.g.,* *MEN1, CDC73*), some genes harbored cumulative LOF SNV frequencies considerably higher than the associated disease phenotype (*e.g.,* *SDHA* and PPGL), indicating a reduced penetrance of such variants. For other genes, (*e.g.,* *NF1*), the apparent high LOF SNV frequency observed was consistent with the known disease prevalence (*e.g.,* NF1 prevalence: 1:3000). Similarly, small indels resulting in an LOF (*i.e.,* frameshift) were absent or very rare in the majority of genes (*i.e.,* 21 of 38 genes harboring ≤1 affected individual), although higher frequencies were observed in several genes in which LOF indels would be anticipated to be disease causing, including *NF1* (~1:5000 individuals), *CASR* (~1:15,000 individuals), *SDHB* (~1:30,000), and *CDC73* (~1:30,000) (Supplemental Table 6). However, it is important to note that the reliability of indel variant calls is most likely reduced compared with SNVs, whereas larger indels will not be identified by capture-based sequencing methods.

### C. Computational Tools Had Low Specificity in Predicting Variant Pathogenicity

**Figure 2.**
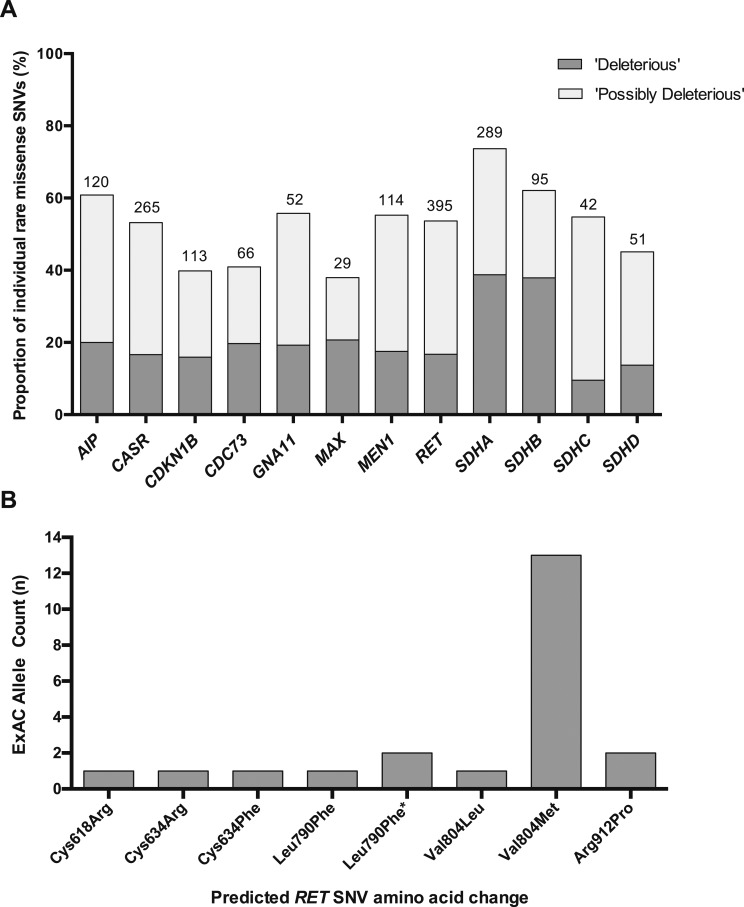
Utility of computational tools to predict rare variant effects and frequency of disease-associated *RET* alleles in the ExAC cohort. (A) Graph shows the proportion of rare SNVs (AF <0.5%) predicted to result in deleterious effects using common computational prediction tools. The number of rare SNVs evaluated for each gene is shown above the respective gene column. In the subset of 12 genes, a mean of 20.5% (range, 9.5% to 37.9%) of gene-specific rare SNVs were categorized as deleterious (*i.e.,* meeting the criteria: AF <0.5%, SIFT <0.05, Polyphen2 probably damaging, and scaled CADD >20), whereas 52.7% (range, 37.9% to 73.7%) were categorized as possibly deleterious (*i.e.,* AF <0.5% and either SIFT ≤0.05 or Polyphen2 probably damaging or possibly damaging). (B) Frequency of *RET* alleles reported as pathogenic in the ExAC cohort. Twenty-two individuals harbored eight different MEN2/familial medullary thyroid cancer‒associated *RET* mutations corresponding to a prevalence of approximately one in 1750 (accounting for incomplete genotyping at the Val804Met locus). Only established MEN2/familial medullary thyroid cancer‒associated *RET* mutations were included in the analysis (Supplemental Table 7). Of these alleles, the majority are classified as moderate risk in the 2015 American Thyroid Association Medullary Thyroid Cancer Guidelines, whereas the two variants predicted to affect the cysteine residue at codon 634 (Cys634Arg, Cys634Phe) are categorized as high risk [27]. The Val804Met variant (observed 13 times) arose in four different ethnic populations. Excluding the Val804Met variant, approximately one in 6000 individuals in the ExAC cohort harbored a pathogenic *RET* mutation. *Two different SNVs predicted a Leu790Phe amino acid substitution.

Computational tools are frequently used to predict the functional effects of missense SNVs on protein function and are often used as an adjunct alongside other clinical and genetic data to provide supporting evidence of variant pathogenicity (*e.g.,* as part of the ACMG algorithm for interpretation of sequence variants). To assess the potential utility of such tools, we analyzed SIFT, Polyphen2, and CADD scores of all rare missense SNVs in a subset of 12 genes. In total, an average of 21% of all rare SNVs in each gene were categorized as deleterious using criteria encompassing all three tools, whereas 53% were described as possibly deleterious (Fig. 2A). The frequency of gene-specific rare missense SNVs classified as deleterious was typically orders of magnitude higher than the prevalence of the associated disorder, indicating that such tools typically have low clinical specificity. For example, ~1:2000 ExAC individuals harbored a rare *MEN1* missense SNV predicted to be deleterious using all three tools, compared with the estimated population prevalence of MEN1 of 1:30,000.

### D. High Prevalence of Disease-Associated Variants in the ExAC Cohort

Having established the frequency and spectrum of rare nonsynonymous SNVs across the 38 genes, we next evaluated the clinical utility of these data. First, we examined the ExAC cohort for variants previously reported as disease-causing in six genes associated with penetrant monogenic disorders (*i.e.,* FHH, MEN1 and MEN2, hyperparathyroidism-jaw tumor syndrome, NF1, and VHL). This analysis revealed that for several genes, the frequency of alleles reported as pathogenic far exceeded the reported prevalence of the associated disease (Supplemental Table 7), giving rise to four possible explanations: The prevalence of disease is higher than reported; prior reports of SNV pathogenicity are incorrect (*i.e.,* variant misclassification); the gene- or allele-specific disease penetrance is lower than reported; or the cohort is unknowingly enriched for hereditary endocrine disorders. For example, approximately one in 1750 individuals was observed to harbor a pathogenic ret proto-oncogene (*RET*) allele (Fig. 2B), compared with an estimated prevalence of MEN2/familial medullary thyroid cancer of approximately one in 80,000 [20]. Thirteen of 22 *RET* mutation carriers harbored the moderate-risk p.Val804Met variant, indicating that this allele is likely associated with low disease penetrance [20–22]. Excluding the p.Val804Met variant, approximately one in 6000 individuals carried moderate- or high-risk *RET* mutations, indicating that the disease penetrance of these alleles may also require reevaluation.

An apparent similar overrepresentation of disease alleles was observed for the calcium sensing receptor (*CASR*), in which approximately one in 3500 individuals harbored a variant previously associated with FHH type 1 (estimated prevalence: 1:15 to 30,000), although in this setting it is plausible that the condition is more prevalent than currently recognized because of its typically asymptomatic phenotype [23]. In contrast, the higher-than-expected occurrence of potentially pathogenic *MEN1* and *VHL* variants in the ExAC cohort indicates that several of these variants were likely misclassified. However, it is important to note that in the case of *VHL*, four of the six likely pathogenic variants occurred in individuals from the TCGA cohort, which included 344 patients with a history of sporadic renal clear cell carcinoma who may be at an increased risk of harboring such germline variants (Supplemental Table 7).

Next, the ExAC cohort was evaluated for individuals harboring clinically-actionable variants in one of seven hereditary endocrine tumor predisposition genes currently included in the ACMG guidelines [19]. Only previously reported pathogenic/likely pathogenic variants were included. This analysis revealed that approximately one in 900 individuals in the ExAC cohort harbored such a clinically actionable variant (Supplemental Table 8). Of note, this collection of disease-associated SNVs did not demonstrate a significant excess of alleles originating from the TCGA cohort (*P* > 0.1) (Supplemental Table 8).

### E. Role of Germline Missense *AIP* Variants in Sporadic Pituitary Tumors

Germline mutations in *AIP* are reported in familial isolated pituitary adenoma kindreds but are associated with reduced penetrance, making it difficult to differentiate between hereditary and sporadic forms [24]. Germline *AIP* variants have also been reported in individuals with apparent sporadic pituitary tumors, although ascribing pathogenicity may be challenging because several *AIP* variants are observed in both disease and control populations [24]. To investigate the role of *AIP* in this setting, we compared the frequencies of rare germline missense *AIP* variants in 1866 individuals with sporadic pituitary adenomas (reported in nine previous studies; Supplemental Table 9) with those observed in the ExAC cohort. Prior analysis demonstrated the predicted frequency of likely cases in the ExAC control population to be negligible (Supplemental Table 10). Of note, only a small excess of rare missense *AIP* variants was observed in the tumor cohort compared with the ExAC cohort (odds ratio, 1.4; confidence interval: 1.0 to 2.0), whereas no overall excess was identified when compared with the European ExAC subpopulation, selected to represent the most relevant cohort for comparison (Table 3). Furthermore, no overrepresentation of several missense *AIP* variants previously reported as pathogenic (*e.g.,* Arg304Gln) were observed in the tumor group, indicating that such variants are most likely benign or associated with very low penetrance (*i.e.,* <1%). A small but notable excess of novel singleton SNVs was observed in the tumor group, predominantly in patients with acromegaly, suggesting genuine enrichment of pathogenic variants in this subgroup (Table 3).

**Table 3.** **Comparison of Frequencies of Missense Germline *AIP* Variants Reported in 1866 Individuals With Sporadic Pituitary Tumors and the ExAC Cohort**

	**Total Missense *AIP* Variants With AF <0.5%** **(n)**	**Arg9Gln** **(n)**	**Arg16His** **(n)**	**Arg304Gln** **(n)**	**Ala299Val** **(n)**	**ExAC Other*****^a^*** **(n)**	**Novel Singleton*****^b^*** **(n)**
All (n = 1866)							
Observed in sporadic pituitary tumor cohorts	38	2	12	9	0	7	8
Predicted from Global ExAC variant frequencies	26.7	0.8	7.3	5.4	1.6	9.4	2.0
Odds ratio (95% CI) observed vs predicted	1.4 (1.0–2.0)	—	1.6 (0.9–2.9)	1.6 (0.8–3.2)	—	—	4.1 (1.9–8.5)

Observed in sporadic pituitary tumor cohorts	38	2	12	9	0	7	8
Predicted from European ExAC variant frequencies	30.2	1.1	11.2	9.1	2.5	4.7	1.6
Odds ratio (95% CI) observed vs predicted	1.2 (0.9–1.7)	—	1.1 (0.6–1.9)	1.0 (0.5–1.9)	—	—	5.2 (2.3–11.4)

Acromegaly (n = 935)							
Observed in acromegaly cohort	18	0	6	5	0	1	6
Predicted from global ExAC variant frequencies	13.2	0.4	3.6	2.7	0.8	4.7	1
Odds ratio (95% CI) observed vs predicted	1.3 (0.8–2.1)	—	—	—	—	—	—

Observed in acromegaly cohort	18	0	6	5	0	1	6
Predicted from European ExAC variant frequencies	15.1	0.5	5.6	4.6	1.2	2.4	0.8
Odds ratio (95% CI) observed vs predicted	1.2 (0.7–1.9)	—	—	—	—	—	—

Prolactinoma (n = 359)							
Observed in prolactinoma cohort	13	1	1	3	0	6	2
Predicted from global ExAC variant frequencies	5.1	0.2	1.4	1	0.3	1.8	0.4
Odds ratio (95% CI) observed vs predicted	2.6 (1.5–4.5)	—	—	—	—	—	—

Observed in prolactinoma cohort	13	1	1	3	0	6	2
Predicted from European ExAC variant frequencies	5.8	0.2	2.1	1.8	0.5	0.9	0.3
Odds ratio (95% CI) observed vs predicted	2.2 (1.3–3.9)	—	—	—	—	—	—

Germline missense variants reported in 1866 individuals (representing 3732 alleles) with apparently sporadic pituitary tumors in whom the *AIP* gene was sequenced. Patient groups represented in the respective studies include those with sporadic child gigantism, sporadic acromegaly presenting in young adulthood and sporadic acromegaly presenting at any age, and individuals with other forms of apparently sporadic pituitary adenomas [including prolactinomas (predominantly macroprolactinomas), nonfunctioning adenomas, and Cushing disease]. This analysis did not include *AIP* sequence analysis from those individuals with apparent familial isolated pituitary adenoma syndromes or those individuals with MEN1-like disorders. A separate subanalysis comparing allele frequencies between the pituitary tumor cohort and just the European subset of the ExAC cohort (n = 33,370 individuals) was performed, deemed to be the most suitable comparator group for the disease cohorts reported in the literature. Estimates of odds ratios and CIs were calculated at http://www.hutchon.net/confidor.htm. For individual variants, odds ratios are provided only where sufficient numbers of alleles were observed to enable meaningful comparison. Some of the studies included in the analysis, reported individuals with unclassified pituitary tumor subtypes. These individuals are not included in the subgroup analysis of prolactinoma and acromegalic cases.

Abbreviation: CI, confidence interval.

^a^ ExAC other: Observed at least once in the ExAC cohort but with an AF <0.5% and excluding R9Q, R16H, R304Q, and A229V variants. *AIP* variants occurring as singletons in ExAC are also reported in this group.

^b^ Novel Singleton: not observed in the ExAC cohort. Predicted number deduced from prevalence of singletons in the ExAC cohort.

### F. Predicting Background Frequencies of Rare Variation Employing Disease-Targeted Gene Panels

Finally, the gene-level estimates of rare SNV frequency were used to model expected rates of background variation likely to be observed when performing simultaneous sequence-analysis of multiple genes (*i.e.,* as occurs with disease-targeted gene panels). Thus, modeling of four disease-targeted gene panels (representing hypercalcemia/parathyroid disorders, pituitary tumors, PPGL, and MEN syndromes) revealed high cumulative estimates of identifying rare variants for each panel (Table 4). For example, the pretest likelihood of identifying at least one rare missense/LOF SNV (AF <0.5%) when employing a 15-gene panel for PPGL was estimated at ~14% (*i.e.,* one in every seven individuals), with ~3% of control individuals harboring a novel singleton variant (*i.e.,* one in every 31 individuals). Such estimates were then modified to allow direct comparison with literature reports. For example, a recent study evaluating 14 genes in patients with sporadic PPGL reported a rare germline mutation frequency of 7%, which did not exceed the background frequency predicted from our analysis of the ExAC cohort [25].

**Table 4.** **Predicted Population-Level Prevalence of Rare Nonsynonymous SNVs Employing Disease-Relevant Gene Panels**

**Disease-Targeted Gene Panel**	**SNV Group:** **AF <0.5%**		**SNV Group:** **AF <0.05%**		**SNV Group:** **Singletons**	
	**Population Prevalence*****^a^*** **(%)**	**Number Needed to Sequence** **(n)**	**Population Prevalence*****^a^*** **(%)**	**Number Needed to Sequence** **(n)**	**Population Prevalence*****^a^*** **(%)**	**Number Needed to Sequence** **(n)**
Pheochromocytoma/paraganglioma *EGLN1,**^b^* *EPAS1,**^b^* *FH, KIF1B,**^b^* *MAX, MEN1, NF1, RET, SDHA, SDHAF2,**^b^* *SDHB, SDHC, SDHD, TMEM127, VHL*	13.9	7.2	8.7	11.6	3.0	32.8
Pituitary tumor *AIP, CDKN1B, GPR101, MEN1, PRKAR1A*	4.9	20.5	1.9	51.3	0.54	185
Hypercalcemia/hyperparathyroid *AP2S1, CASR, CDC73, CDKN1A, CDKN1B, CDKN2B, CDKN2C, GNA11, MEN1, RET*	8.1	12.3	3.8	26.6	1.3	77.9
Multiple endocrine neoplasia *CDKN1B, MEN1, NF1, PRKAR1A, RET, VHL*	6.3	15.8	3.8	26.1	1.4	70.8

Hypothetical gene panel to include sequencing of coding region of all genes listed under each respective heading. The number needed to sequence represents an estimation of the average number of individuals requiring sequencing to identify a variant in each relevant group.

^a^ Calculation based on occurrence of a rare variant (of the relevant frequency classification) in at least one of the genes in the respective panel.

^b^ Germline variants reported in a single or a very low number of individuals/kindreds.

## 3. Discussion

The accurate interpretation of germline genetic variants is essential to provide appropriate patient care but remains imprecise and may lead to diagnostic uncertainty [6, 7]. Recent studies have highlighted the occurrence of widespread variant misclassification in association with several disorders, which have frequently arisen as a result of ascertainment and reporting bias, a failure to genetically characterize sufficiently large patient and control cohorts, and an overreliance on SNV rarity *per se* and computational tools in predicting pathogenicity [7–9]. Furthermore, inaccurate estimates of disease penetrance are also widespread, as illustrated by recent reports of apparently healthy individuals carrying large numbers of disease-associated alleles [14, 15, 26].

In this study, we used the ExAC cohort to quantify the spectrum and frequency of rare nonsynonymous germline variants occurring in a broad range of genes associated with hereditary endocrine diseases and illustrate the potential utility of this information for improved variant interpretation, both in ascribing potential pathogenicity and in reevaluating estimates of disease penetrance. Of note, we observed marked differences in the frequency or rare nonsynonymous SNVs between genes after correcting for coding-region length, and this was correlated with the degree of evolutionary conservation. The observation that the lowest rare SNV frequencies were observed in genes with the highest degrees of evolutionary conservation is not unexpected, as several of the most highly conserved genes regulate essential cellular functions (*e.g.,* *CDC73*, *AP2S1*, and *PRKAR1A*). Therefore, these genes are likely to be under strong evolutionary selection pressure to conserve key cellular processes, thereby resulting in a relative intolerance to variation.

**Figure 3.**
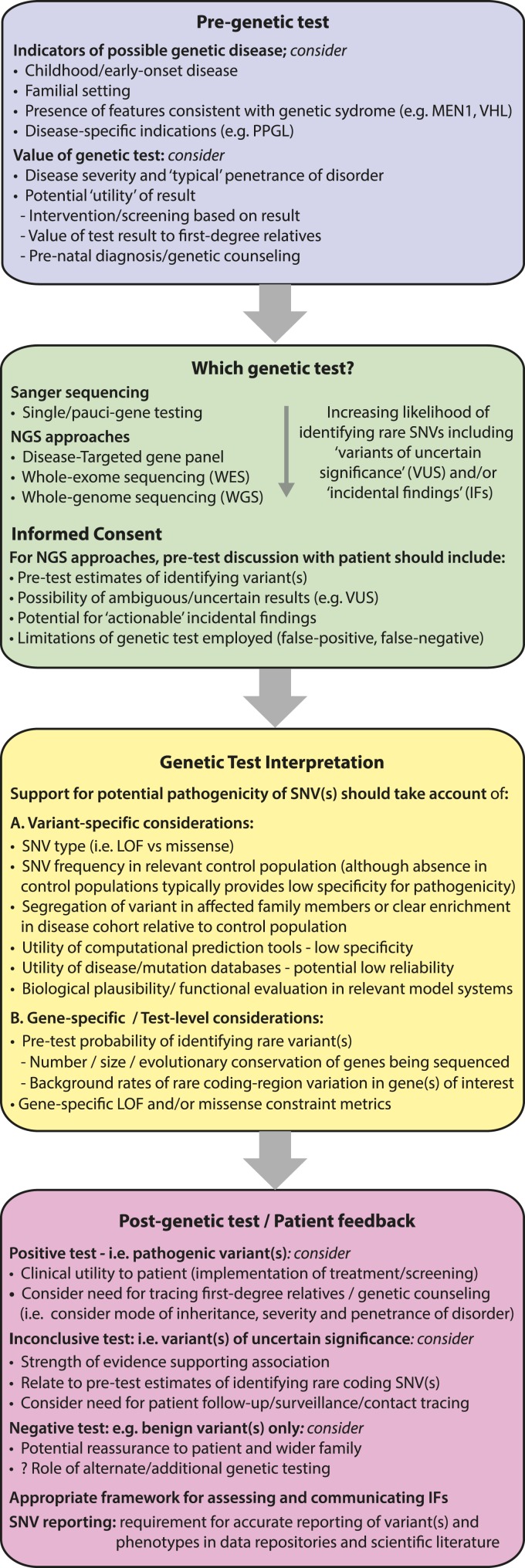
Illustrative workflow outlining considerations for genetic testing and variant interpretation in clinical and research settings. Evaluating the spectrum and frequency of rare variations in large population cohorts may enhance the process of genetic testing at different stages of the clinical workflow. For example, as the content of genetic testing increases, the likelihood of identifying rare coding variants increases (*e.g.,* VUSs and IFs). The current study illustrates how gene-specific cumulative rare variant frequencies may be used to establish pretest estimates for identifying such variants. This information could be incorporated into the informed consent process to alert patients to the likelihood of ambiguous test results. Although guidelines exist to standardize the process of ascribing pathogenicity to germline variants, these typically focus on variant-specific features. The current study highlights how gene-level factors, including estimates of rare missense/LOF variation together with metrics of constraint, may aid variant classification. For example, when the cumulative frequency of LOF variants in the control population exceeds the prevalence of the disease under investigation, the likelihood that such a variant is a high penetrance disease-allele is substantially reduced. Conversely, variants in genes with very low rare variation burden potentially have a higher likelihood of pathogenicity. In the future, it is possible that this information may contribute to Bayesian models for disease in which the likelihood of variant pathogenicity and/or disease expression is adjusted according to clinical factors as well as gene- and variant-level metrics. Furthermore, for potential disease alleles, refined estimates of disease penetrance may be established by evaluating the frequency of the variant in disease and control populations, and such accurate estimates are essential for appropriate genetic counseling (*e.g.,* to determine the value of implementing treatment/surveillance programs and/or screening of first-degree relatives). In addition, when ambiguous test results have been obtained (*e.g.,* VUSs), further refinement of risk may be established by relating the test result to both the pretest estimate of detecting such variation and constraint metrics, which together may aid the clinician and patient in making informed decisions regarding future care. Finally, these studies illustrate the need for transparent and accurate reporting of genetic data coupled to phenotypes (*i.e.,* avoiding positive reporting bias) to improve the accuracy of existing disease/mutation databases. IF, incidental finding; NGS, next-generation sequencing; VUS, variant of uncertain significance.

Although consensus guidelines have been established by the ACMG for the clinical interpretation of germline sequence variants, in many instances an unambiguous assignment of pathogenicity is not possible [6]. In the absence of strong supporting evidence for pathogenicity (*e.g.,* identification of the LOF allele in genes in which LOF is known to result in disease), current analyses often use a combination of variant-level features, such as the absence of a variant in a control population, as well as predictions from computational tools [6]. Although these approaches in isolation do not provide sufficient evidence to categorize variants as pathogenic/likely pathogenic (*i.e.,* enabling categorization only as a variant of uncertain significance), they are frequently cited as supporting evidence of pathogenicity and may ultimately result in a patient being managed as if the variant is disease causing. Our analyses indicate that variant rarity (*i.e.,* the absence or very low AF of a variant in a control database) together with computational prediction tools frequently have a low specificity for ascribing clinically relevant effects and that relying on such features likely overestimates pathogenicity. For example, we observed that across the 38 genes, the majority of individual variants were observed only once in the ExAC cohort and that a similar majority of rare SNVs were classified as potentially deleterious by at least one of the computational tools evaluated. However, our studies also demonstrated how the use of additional gene-specific factors, including cumulative rare variant frequencies together with metrics of missense and LOF constraint, may provide important additional context when incorporated into the variant interpretation workflow (Fig. 3). For example, variants in genes associated with very low rates of rare variation and intolerant constraint metrics are more likely to be pathogenic than those in genes with greater tolerance of variation, and such information may be useful to the clinician in deciding how to counsel/follow-up a patient with an ambiguous test result (*e.g.,* a variant of uncertain significance). Indeed, it is plausible that in the future such gene-specific constraint metrics and/or estimates of rare variant burden may be incorporated into bioinformatic or clinical computational algorithms to improve estimates of variant pathogenicity and/or disease penetrance.

Furthermore, we demonstrated how quantifying the burden of rare variation in a given set of genes may be used to derive an expected frequency of background rare variants that should be anticipated when using disease-targeted gene panels. Of note, these studies illustrate how a failure to consider the high frequency of background rare variation arising from the use of such gene panels is likely to contribute to diagnostic uncertainty and may have confounded earlier genetic studies.

In the current study, we observed improbably high frequencies of variants reported as pathogenic in several genes (*e.g.,* *RET*, *VHL*, and *MEN1*), indicating their likely prior misclassification and/or overestimates of disease penetrance. Indeed, the need to define allele-specific estimates of disease penetrance is an important concept, as recently illustrated for prion disease in which the disease-penetrance of individual *PRNP* variants ranged from <0.1% to ~100% [15]. Our studies suggest that similar dynamic ranges of penetrance are likely to occur for alleles associated with endocrine disease (*e.g.,* *RET*), and quantifying these is essential to enable appropriate patient care (*e.g.,* appropriate guidance on the timing/requirement for prophylactic thyroidectomy in individuals with *RET* variants associated with MEN2/familial medullary thyroid cancer [27].

However, differentiating benign from low-penetrance alleles remains challenging, even with large disease and control cohorts [15]. For example, the failure to demonstrate enrichment of several individual *AIP* variants (*e.g.,* Arg304Gln) in a large pituitary tumor cohort compared with the ExAC population cannot exclude an etiological role in disease, although it suggests that any disease relationship is associated with extremely low penetrance and that the overwhelming majority of such variant carriers will not manifest clinical features. Indeed, establishing accurate estimates of disease penetrance should be a priority, and it is evident that for several hereditary endocrine genes, penetrance may have been overstated (*e.g.,* *SDHx* genes) because of unintentional ascertainment bias and/or the inclusion of index cases in such estimates [28].

However, for accurate estimates of penetrance to be established, it is essential that the control population used be closely matched to the study population, thereby avoiding confounding from population-specific differences in variant frequencies (*e.g.,* the presence of founder mutations in local populations). For example, although ExAC offers large numbers of individuals from specific ethnic groups (*e.g.,* European ancestry), other populations are underrepresented, and for these groups, it may not provide a suitable comparator group.

Periodic clinical, biochemical, and/or radiological screening is generally recommended for carriers of pathogenic alleles associated with hereditary endocrine tumor syndromes (*e.g.,* *MEN1, RET,* and *SDHB*) [17, 27, 29]. Our results reveal that approximately one in 900 individuals in the ExAC cohort harbored an apparent clinically actionable missense/LOF SNV in one of seven endocrine tumor predisposition genes currently included in the ACMG guidelines for the reporting of incidental genetic findings in clinical exome and genome data, which is considerably higher than the combined prevalence of the associated disorders (estimated to be 1:10,000). Furthermore, the approximately one in 900 figure likely represents an underestimate because the current analysis was limited to SNVs reported as pathogenic in existing databases and excluded other potentially deleterious alleles, including indels, which were observed in a small number of additional individuals (*e.g.,* in *SDHB* and *SDHD*). This not only highlights the potential clinical burden that increased genetic testing may bring but also emphasizes the need for accurate estimates of variant pathogenicity and penetrance because the potential for patient harm arising through tumor surveillance programs is not insignificant [19, 30]. Furthermore, it is plausible that future decisions regarding the implementation of screening protocols may be determined by defined thresholds of variant penetrance.

Our analysis has several potential limitations. First, although not knowingly enriched for hereditary endocrine disorders, the ExAC cohort will contain individuals with these diseases as well as controls with polygenic disorders with accompanying disease-associated alleles. However, the inclusion of a broad range of genes and associated conditions reduced the likelihood of widespread enrichment for relevant disease phenotypes. For example, one potential concern was that the inclusion of germline samples from the TCGA cohort might result in an overrepresentation of alleles in genes associated with hereditary endocrine tumors; although this cohort does not include endocrine tumors relevant to the genes under study (*e.g.,* PPGL, medullary thyroid cancer), it was reassuring that the TCGA samples did not contribute an excess of rare SNVs or likely pathogenic alleles in the genes investigated, with the possible exception of *VHL*, in which the inclusion of renal carcinoma cases may have introduced a risk of bias.

A further limitation of the current study is that individual rare variants were considered in isolation in our analysis, and potential interactions between variants or other modifying influences (*e.g.,* *cis*-acting elements) could not be evaluated. In addition, high-quality SNV calls were assumed to be accurate, and although the performance of the ExAC variant calling pipelines has been extensively validated (*e.g.,* SNV sensitivity of 99.8% and false discovery rate of 0.06%), confirmatory sequencing of individual variants was not undertaken. Furthermore, caution is required in interpreting SNVs in regions with reduced sequence coverage (*e.g.,* exon 1 of *VHL*) or those presenting difficulties in the sequencing pipelines. For example, the reliability of sequence data for genes with multiple pseudogenes (*e.g.,* *SDHA*) may be reduced, although in these instances, visual inspection of individual sequence reads covering regions adjacent to the SNVs enabled increased confidence in the variant call (Supplemental Fig. 3).

Another limitation of our study is that we excluded indels from the main analysis because of their reduced reliability of detection [31]; although we quantified the frequency of small LOF indels in a separate analysis, the accuracy of such estimates may be reduced compared with SNVs. Of note, the current study could not evaluate the frequency of medium- and large-sized indels because the current methodology does not detect such changes, although future studies employing whole-genome sequencing may help to address these deficiencies. Finally, it is important to note that our study has two other limitations. First, the analysis was limited to the 38 genes selected, and it is possible that additional genes not included in this list may contribute to some of the clinical phenotypes, thereby reducing the accuracy of estimates of disease prevalence and/or penetrance. Second, the ExAC data set does not allow an evaluation of epigenetic changes that may contribute to specific disease phenotypes (*e.g.,* alterations in methylation at the *GNAS* locus in pseudohypoparathyroidism Ib).

In summary, these studies demonstrate how quantifying rare germline variations in a large control cohort such as ExAC may be exploited to improve variant interpretation and clinical decision-making. Furthermore, this information may be incorporated into different stages of the clinical genetic testing workflow (Fig. 3) and may provide important context when communicating uncertain test results to patients. Finally, as genetic testing moves increasingly into the mainstream, these studies highlight the need for increased vigilance in the undertaking and reporting of genetic studies to improve estimates of variant pathogenicity and penetrance, thereby enabling clinicians and patients to make informed decisions regarding their care.
