# Supplementary material for: Utility of Population-Level DNA Sequence Data in the Diagnosis of Hereditary Endocrine Disease
Source: J Endocr Soc. 2017 Nov 15;1(12):1507–26. doi: 10.1210/js.2017-00330 (PMC5740525; doi:10.1210/js.2017-00330)
Supplement: Supplementary file 1 [file js-01-12-1507.st1.docx]

**SUPPLEMENTARY APPENDIX**

**Utility of Population-Level DNA sequence Data in the Diagnosis of Hereditary Endocrine Disease**

**List of authors**

Paul J Newey, Jonathan Berg, Kaixin Zhou, Colin NA Palmer, Rajesh V Thakker

**Supplemental Tables**

**Supplemental Table 1. Tumor sample subtypes included from The Cancer Genome Atlas (TCGA) subgroup in ExAC cohort**

| **TCGA Tumor subgroup (germline samples)** | **Samples (n)** |
| --- | --- |
| Adrenocortical carcinoma | 78 |
| Bladder Urothelial Carcinoma | 262 |
| Brain Lower Grade Glioma | 458 |
| Breast invasive carcinoma | 965 |
| Cervical squamous cell carcinoma and endocervical adenocarcinoma | 149 |
| Colon adenocarcinoma | 335 |
| Esophageal carcinoma | 17 |
| Glioblastoma multiforme | 314 |
| Head and Neck squamous cell carcinoma | 506 |
| Kidney Chromophobe | 62 |
| Kidney renal clear cell carcinoma | 344 |
| Kidney renal papillary cell carcinoma | 173 |
| Liver hepatocellular carcinoma | 180 |
| Lung adenocarcinoma | 531 |
| Lung squamous cell carcinoma | 367 |
| Ovarian serous cystadenocarcinoma | 276 |
| Pancreatic adenocarcinoma | 112 |
| Prostate adenocarcinoma | 411 |
| Rectum adenocarcinoma | 109 |
| Sarcoma | 115 |
| Skin Cutaneous Melanoma | 416 |
| Stomach adenocarcinoma | 369 |
| Thyroid carcinoma (differentiated)* | 486 |
| Uterine Carcinosarcoma | 57 |
| Uterine Corpus Endometrial Carcinoma | 509 |
| **Total** | **7601** |

*No cases of medullary thyroid cancer (MTC) are included in this subgroup

Each of the contributing TCGA samples in ExAC represent germline blood samples (i.e. not tumor samples)

TCGA samples (n=7,601) comprise 12.5% of the total ExAC cohort (n=60,706)

**Supplemental Table 2: Estimates of evolutionary conservation of genes in study. Pairwise comparison scores of ortholog amino acid sequence identity relative to the human protein sequence**

| **Gene** | ***P.troglodytes***  *Chimpanzee* | ***M.mulatta***  *Rhesus Macaque* | ***C.lupus***  *Wolf* | ***B.taurus***  *Cow* | ***M.musculus***  *Mouse* | ***R.norvegicus***  *Rat* | ***G.gallus*** *Chicken* | ***X.tropicalis***  *Clawed Frog* | ***D.rerio***  *Zebrafish* |
| --- | --- | --- | --- | --- | --- | --- | --- | --- | --- |
| *AP2S1* | 100 | 100 | 100 | 100 | 100 | 100 | 99.1 | 99.3 | 99.3 |
| *PRKAR1A* | 100 | 99.7 | 97.9 | 97.9 | 96.9 | 97.4 | 92.7 | 92.6 | 91 |
| *THRB* | 100 | 99.8 | 98.5 | 96.1 | 96.5 | 96.3 | 97.3 | 92.8 | 91 |
| *CDC73* | 100 | 100 | 100 | 100 | 100 | 99.8 | 97.7 | 94 | 90.9 |
| *GNA11* | 93.5 | 99.2 | - | 98.6 | 98.1 | 96.7 | 97.2 | 92.8 | 90.8 |
| *KIF1B* | - | 97.9 | 97.8 | 97.3 | 97.1 | 96.8 | 95 | 90.2 | 88.4 |
| *NF1* | 100 | 99.1 | 99.4 | - | 98.5 | 98.6 | 95.5 | 92.1 | 88.4 |
| *FH* | 99.4 | 98.4 | 95.7 | 95.3 | 93.3 | 93.3 | 85.6 | 88.4 | 87.8 |
| *TMEM127* | 100 | 100 | 100 | 99.6 | 98.7 | 99.2 | 95.7 | 89.8 | 87.1 |
| *THRA* | 100* | 100* | 99.8 | 99.2 | 99.3 | 99.3 | 90.4 | 88.5 | 85.3 |
| *GNAS* | 99.4 | 95 | 95.1 | 94.8 | 78 | 78.1 | 92.2 | 86.7 | 85.1 |
| *MAX* | 100 | 100 | 100 | 99.4 | 99.4 | 98.8 | 95 | 93.7 | 85 |
| *SDHB* | 100 | 98.2 | 95.4 | 93.9 | 92.4 | 91.7 | 90.2 | 90.3 | 84.7 |
| *SDHA* | 99.4 | 89.3 | 95.8 | 91.1 | 94.9 | 94.8 | 89.2 | 87.8 | 83.4 |
| *GATA3* | 99.8 | 98.6 | 96.8 | 96.4 | 96.6 | 96.4 | 92.3 | 82.2 | 81.5 |
| *CHD7* | 99.9 | 99.4 | 95.9 | 92.5 | 95.1 | 94.8 | 92.4 | 86.6 | 76.5 |
| *CASR* | 99.4 | 98.5 | 94.3 | 93.7 | 93.5 | 93.3 | 87.9 | 78.7 | 76 |
| *EGLN1* | 99.3 | 97.7 | 83 | - | 82.4 | 89.3 | 87.6 | 80.8 | 75.8 |
| *KCNJ5* | 99 | 98.6 | 94 | 93.8 | 94.3 | 93.8 | 86.2 | 84.1 | 75.4 |
| *MEN1* | 99.7 | 100 | 98.4 | 98.7 | 96.7 | 97 | - | 73.9 | 72.6 |
| *AIP* | 99.4 | 97.9 | 94.5 | 93.6 | 94.2 | 93.9 | 79.9 | 66.8 | 66.8 |
| *PHEX* | 99.5 | 98.7 | 95.2 | 94.3 | 96 | 95.5 | 82 | 70.6 | 65.9 |
| *SDHC* | 98.2 | 98.8 | 98.8 | 91.7 | 89.9 | 79.9 | 82.2 | 74.6 | 63.6 |
| *EPAS1* | 99.9 | 97.7 | 90.1 | 87.7 | 88 | 86 | 76.3 | 67.1 | 63.1 |
| *SDHAF2* | 98.8 | 98.8 | 89.6 | 90.3 | 92 | 91.5 | 70.3 | 62 | 62.1 |
| *RET* | 95 | 94.8 | 86.9 | 87.4 | 83.5 | 85.4 | 67.5 | 63.1 | 61.5 |
| *SDHD* | 98.7 | 96.2 | 86.2 | 87.2 | 82.4 | 81.1 | 66.4 | 69.1 | 61 |
| *CDKN1B* | 100 | 98.5 | 90.4 | 90.9 | 87.8 | 87.2 | 68.7 | - | 60.2 |
| *KAL1* | 96.9 | 94.4 | 84.5 | 83.4 | - | - | 77.5 | 73 | 58.6 |
| *VHL* | 98* | 98 | 89.6 | 93.9 | 86.4 | 93 | 71.6 | 66.2 | 54.1 |
| *CDKN2C* | 100 | 100 | 95.2 | 94 | 92.3 | 92.9 | 80.9 | 63 | 53.9 |
| *GPR101* | 99.6 | 96.4 | 85.6 | 87.4 | 72.6 | 72.8 | - | 53.7 | 47.5 |
| *ARMC5* | 94* | 95.7 | 89.2 | 88.9 | 86.8 | 85.8 | - | 46.8 | 44.2 |
| *CDKN2B* | 93.5 | 98.6 | 83.3 | 86.3 | 88.3 | 88.3 | 63.9 | - | 44* |
| *GHR* | 98.9 | 94.2 | 82.3 | 77.3 | 70 | 70.2 | 62.3 | 49.6 | 43.8 |
| *CDKN1A* | 100 | 97 | 82.9 | 82 | 79.2 | 76.5 | - | - | 42* |
| *FGF23* | 98 | 96.4 | 80.5 | 79.4 | 70.5 | 71.7 | 58.8 | 57.7 | 41.4 |
| *PRLR* | 100 | 96.4 | 75.4 | 69.8 | 69.8 | 70.2 | 51.3 | 49.9 | 40.2 |

Pairwise comparison scores obtained for each gene at NCBI Homologene (http://www.ncbi.nlm.nih.gov/homologene). In each case the amino acid sequence identify percentage compared to the reference human sequence is reported.

*Where data was not available at HomoloGene, missing data points were extrapolated from orthologs identified in Ensembl (http://www.ensembl.org). In some instance no relevant ortholog/data was available (marked with (-)). Data arranged by reducing conservation in *Danio rerio. (*Data accessed: March-June 2016).

**Supplemental Table 3: Missense and Loss of function (LOF) constraint metrics**

| **Gene** | **Missense SNVs*** | | | **Loss of Function (LOF) SNVs** | | |
| --- | --- | --- | --- | --- | --- | --- |
|  | **Expected** | **Observed** | **Z Score** | **Expected** | **Observed** | **pLI Score** |
| ***AIP*** | 143 | 116 | 1.12 | 12 | 2 | 0.67 |
| ***AP2S1*** | 63.7 | 5 | 3.6 | 7.8 | 0 | 0.92 |
| ***ARMC5*** | 428.7 | 288 | 3.32 | 19.6 | 6 | 0.05 |
| ***CASR*** | 402.6 | 258 | 3.52 | 26.3 | 6 | 0.37 |
| ***CDC73*** | 178.4 | 66 | 4.12 | 32.7 | 1 | 1 |
| ***CDKN1A*** | 79.2 | 79 | 0.01 | 5.5 | 3 | 0.03 |
| ***CDKN1B*** | 97.6 | 111 | -0.66 | 5.9 | 0 | 0.85 |
| ***CDKN2B*** | 81.8 | 64 | 0.96 | 3.5 | 1 | 0.28 |
| ***CDKN2C*** | 60.5 | 45 | 0.98 | 3.9 | 1 | 0.32 |
| ***CHD7*** | 899.8 | 782 | 1.92 | 87.7 | 2 | 1 |
| ***EGLN1*** | 209.9 | 75 | 4.55 | 12.4 | 1 | 0.93 |
| ***EPAS1*** | 304.5 | 314 | -0.27 | 26.2 | 2 | 1 |
| ***FGF23*** | 119.2 | 102 | 0.77 | 5.5 | 3 | 0.03 |
| ***FH*** | 147.5 | 129 | 0.75 | 14.3 | 4 | 0.15 |
| ***GATA3*** | 188.9 | 106 | 2.95 | 11.1 | 1 | 0.88 |
| ***GHR*** | 177.8 | 213 | -1.29 | 18.6 | 9 | 0 |
| ***GNA11*** | 191 | 52 | 4.92 | 10.2 | 0 | 0.97 |
| ***GNAS*** | 424.7 | 242 | 4.34 | 26.2 | 1 | 1 |
| ***GPR101*** | 141.1 | 102 | 1.61 | 4.8 | 1 | 0.4 |
| ***KAL1*** | 143 | 123 | 0.82 | 21.8 | 3 | 0.94 |
| ***KCNJ5*** | 167.8 | 129 | 1.46 | 8 | 2 | 0.31 |
| ***KIF1B*** | 625.7 | 419 | 4.04 | 82.9 | 4 | 1 |
| ***MAX*** | 67.2 | 29 | 2.28 | 9.3 | 0 | 0.95 |
| ***MEN1*** | 274 | 112 | 4.79 | 20 | 0 | 1 |
| ***NF1*** | 850 | 480 | 6.22 | 104.9 | 14 | 1 |
| ***PHEX*** | 171.6 | 137 | 1.29 | 26.9 | 1 | 1 |
| ***PRKAR1A*** | 139.8 | 55 | 3.51 | 20.4 | 0 | 1 |
| ***PRLR*** | 180 | 203 | -0.84 | 16.5 | 2 | 0.92 |
| ***RET*** | 469.6 | 391 | 1.77 | 34.4 | 2 | 1 |
| ***SDHA*** | 280 | 201 | 2.32 | 27.7 | 13 | 0 |
| ***SDHAF2*** | 56.8 | 56 | 0.05 | 5.4 | 6 | 0 |
| ***SDHB*** | 93 | 93 | 0.02 | 15.5 | 5 | 0.05 |
| ***SDHC*** | 59.2 | 42 | 1.1 | 8.6 | 3 | 0.1 |
| ***SDHD*** | 51.5 | 50 | 0.1 | 8 | 1 | 0.71 |
| ***THRA (canonical)*** | 221.3 | 90 | 4.32 | 19 | 5 | 0.17 |
| ***THRB*** | 169.6 | 94 | 2.84 | 15.2 | 0 | 0.99 |
| ***TMEM127*** | 94.1 | 72 | 1.12 | 4.7 | 2 | 0.1 |
| ***VHL*** | 92 | 77 | 0.76 | 6 | 3 | 0.03 |

Constraint metric scores for each gene were obtained directly from the ExAC browser (http://exac.broadinstitute.org/). For LOF analysis, only SNVs resulting in nonsense amino acid changes of those directly affecting splice donor or splice acceptor sites are included. For both missense and LOF SNVs, the number of predicted and observed variants reflects unique variants (i.e. does not take into account multiple observations of the same allele).

**Supplemental Table 4. Comparison of rare SNV frequency in global and non-TCGA ExAC cohorts**

|  | **SNV group:**  **Allele Frequency (AF) <0.5%** | | **SNV group:**  **Allele Frequency (AF) <0.05%** | | **SNV group:**  **Singleton** | |
| --- | --- | --- | --- | --- | --- | --- |
| **Gene^§^** | **Global ExAC Number Needed to Sequence (n)** | **Non-TCGA ExAC Number Needed to Sequence (n)** | **Global ExAC Number Needed to Sequence (n)** | **Non-TCGA ExAC Number Needed to Sequence (n)** | **Global ExAC Number Needed to Sequence (n)** | **Non-TCGA ExAC Number Needed to Sequence (n)** |
| ***CDC73*** | 468 | 501 | 468 | 501 | 1208 | 1327 |
| ***CDKN1B*** | 109 | 108 | 263 | 270 | 1123 | 1179 |
| ***MEN1*** | 253 | 278 | 270 | 297 | 658 | 705 |
| ***NF1*** | 47 | 48 | 66 | 70 | 186 | 202 |
| ***PRKAR1A*** | 432 | 421 | 828 | 856 | 1514 | 1609 |
| ***RET*** | 34 | 37 | 71 | 80 | 231 | 258 |
| ***SDHA*** | 78 | 77 | 110 | 111 | 516 | 540 |
| ***SDHAF2*** | 227 | 227 | 502 | 491 | 1890 | 1713 |
| ***SDHB*** | 168 | 171 | 319 | 329 | 1048 | 1206 |
| ***SDHC*** | 625 | 610 | 625 | 610 | 2757 | 2529 |
| ***SDHD*** | 433 | 421 | 659 | 663 | 2245 | 2308 |
| ****VHL*** | 138 | 261 | 206 | 379 | 429 | 1077 |
| ***VHL (exons 2 & 3)*** | 481 | 505 | 509 | 569 | 2618 | 2794 |

§ Genes associated with hereditary endocrine tumor syndromes were selected for inclusion in this analysis

The Number Needed to Sequence (NNS) equates to the mean number of individuals (reported to the nearest whole number) requiring sequencing to identify a rare variant of each type (i.e. AF <0·5%, AF <0·05% or singleton).

* It is important to note that minor differences in variant filtering parameters between the global and non-TCGA ExAC cohorts prevent direct comparison between genes with regions of low sequence coverage (i.e. *VHL*). Thus, although differences in rare variant frequencies are observed between global and non-TCGA ExAC cohorts for *VHL*, this is due to poor sequence coverage of exon 1 rather than enrichment for *VHL* rare variants in the TCGA samples. For example, similar variant frequencies are observed when the analysis is limited to VHL exons 2 and 3, which have near complete sequence coverage. The reduced reliability of the rare variant frequency estimates for *VHL* is stated throughout the manuscript.

**Supplemental Table 5: Cumulative loss of function (LOF) SNV allele frequencies in ExAC cohort**

| **Gene** | **Cumulative**  **LOF SNV Population Frequency (%)** | **Number Need to**  **Sequence (NNS)** |
| --- | --- | --- |
| ***AP2S1*** | 0 | (>60,700) |
| ***CDKN1B*** | 0 | (>60,700) |
| ***GNA11*** | 0 | (>60,700) |
| ***GPR101^§^*** | 0 | (>60,700) |
| ***MAX*** | 0 | (>60,700) |
| ***MEN1*** | 0 | (>60,700) |
| ***PRKAR1A*** | 0 | (>60,700) |
| ***THRB*** | 0 | (>60,700) |
| ***CDC73*** | 0.0016 | 60400 |
| ***CDKN2C*** | 0.0016 | 60700 |
| ***EGLN1*** | 0.0016 | 60700 |
| ***RET*** | 0.0016 | 60400 |
| ***SDHD*** | 0.0016 | 60700 |
| ***PHEX^§^*** | 0.0023 | 43800 |
| ***GATA3*** | 0.0033 | 30300 |
| ***PRLR*** | 0.0033 | 30200 |
| ***AIP*** | 0.0034 | 29150 |
| ***CDKN2B*** | 0.0043 | 23100 |
| ***EPAS1*** | 0.0049 | 20200 |
| ***KCNJ5*** | 0.0049 | 20200 |
| ***TMEM127**** | 0.0049 | 20200 |
| ***CDKN1A*** | 0.0051 | 19700 |
| ***CHD7*** | 0.0051 | 19700 |
| ***FGF23*** | 0.0051 | 19700 |
| ***KIF1B*** | 0.0066 | 15200 |
| ***KAL1^§^*** | 0.0097 | 10300 |
| ***SDHC*** | 0.0099 | 10100 |
| ***SDHB*** | 0.012 | 8640 |
| ***CASR*** | 0.013 | 7580 |
| ***VHL**** | 0.013 | 7470 |
| ***ARMC5*** | 0.014 | 7250 |
| ***FH*** | 0.018 | 5360 |
| ***SDHAF2*** | 0.020 | 5040 |
| ***THRA_1*** | 0.020 | 5050 |
| ***GHR*** | 0.020 | 4940 |
| ***GNAS*** | 0.021 | 4700 |
| ***NF1*** | 0.034 | 2960 |
| ***THRA_2*** | 0.043 | 2310 |
| ***SDHA±*** | 0.094 | 1060 |

Only SNVs predicted to result in a LOF were considered including those predicted to result in nonsense amino acid changes, or those directly affecting acceptor or donor splice sites. Insertion and/or deletions were excluded from analysis. In contrast to the LOF constraint metric pLI, the cumulative LOF allele frequencies account for multiple observations of individual alleles.

^§^X-linked – frequency estimates in female (i.e. diploid);

*Reduced coverage of some exonic regions may reduce reliability of results (e.g. *VHL*). Abbreviations: LOF, loss of function.

***±*** Visual inspection and BLAST searching of the recurrent LOF alleles in *SDHA* indicated that these were unlikely to be due to mapping problems resulting from known *SDHA* pseudogenes.

Abbreviations: LOF, loss of function; NNS, number need to sequence

**Supplemental Table 6: Cumulative loss of function (LOF) indel frequencies in ExAc cohort**

| **Gene** | **No. Unique Indels §** | **Cumulative Population LOF indel frequency (%)** | **Estimated indel carrier frequency in ExAC population**  **NNS** | **Relevant Disease reported to be associated with indels±** |
| --- | --- | --- | --- | --- |
| ***AIP*** | 0 | 0 | (>60,700) | Yes |
| ***AP2S1*** | 0 | 0 | (>60,700) | - |
| ***CDKN1A*** | 0 | 0 | (>60,700) | - |
| ***CHD7*** | 0 | 0 | (>60,700) | Yes |
| ***EPAS1*** | 0 | 0 | (>60,700) | - |
| ***FGF23*** | 0 | 0 | (>60,700) | - |
| ***GATA3*** | 0 | 0 | (>60,700) | Yes |
| ***GPR101*** | 0 | 0 | (>60,700) | - |
| ***KAL1*** | 0 | 0 | (>60,700) | Yes |
| ***MAX*** | 0 | 0 | (>60,700) | Yes |
| ***MEN1*** | 0 | 0 | (>60,700) | Yes |
| ***PHEX*** | 0 | 0 | (>60,700) | Yes |
| ***SDHC*** | 0 | 0 | (>60,700) | Yes |
| ***VHL*** | 0 | 0 | (>60,700) | Yes |
| ***EGLN1*** | 1 | 0.00016 | 60,700 | - |
| ***SDHD*** | 1 | 0.00016 | 60,700 | Yes |
| ***TMEM127*** | 1 | 0.00016 | 60,700 | Yes |
| ***CDKN2C*** | 1 | 0.00016 | 60,600 | - |
| ***RET*** | 1 | 0.00016 | 60,600 | No* |
| ***PRKAR1A*** | 1 | 0.00016 | 60,400 | Yes |
| ***THRB*** | 1 | 0.0033 | 30,300 | Yes |
| ***CDC73*** | 2 | 0.0033 | 30,300 | Yes |
| ***KCNJ5*** | 2 | 0.0033 | 30300 | - |
| ***PRLR*** | 2 | 0.0033 | 30300 | - |
| ***SDHB*** | 2 | 0.0033 | 30200 | Yes |
| ***CDKN1B*** | 2 | 0.0033 | 30000 | Yes |
| ***GNA11*** | 1 | 0.0035 | 28,500 | - |
| ***ARMC5*** | 2 | 0.0036 | 27,200 | - |
| ***CDKN2B*** | 2 | 0.0041 | 24700 | - |
| ***SDHA*** | 2 | 0.0049 | 20200 | Yes |
| ***SDHAF2*** | 3 | 0.005 | 20200 | - |
| ***KIF1B*** | 3 | 0.005 | 20100 | - |
| ***GHR*** | 3 | 0.0066 | 15200 | - |
| ***FH*** | 4 | 0.0066 | 15200 | Yes |
| ***CASR*** | 4 | 0.0066 | 15200 | Yes |
| ***GNAS*** | 2 | 0.007 | 14300 | Yes |
| ***THRA*** | 2 | 0.008 | 11700 | - |
| ***NF1*** | 11 | 0.028 | 3540 | Yes |

§Each of the indels identified were of small size (involving ≤7 nucleotides). The majority (>90%) involved indels of ≤3 nucleotides. The number of unique indels/gene is noted although in some instances indels were observed multiple times. Only high-confidence variant calls were included in the analysis.

*LOF *RET* mutations are not associated with MEN2, but are observed in a proportion of individuals with Hirschsprung Disease.

Abbreviation: NNS, Number Needed to Sequence

Abbreviations: LOF, loss of function; indels, insertions or deletions; NNS, number need to sequence

**Supplemental Table 7; Prevalence of ‘pathogenic’ SNVs in penetrant monogenic endocrine disease genes in ExAC cohort**

| **Monogenic disorder** | **Gene** | **Estimated population prevalence / live birth rate** | **Proportion of affected individuals with mutation in relevant gene(s)** | **Typical penetrance of disease in mutation carriers** | **Prevalence of LOF SNVs in ExAC** | **Prevalence of rare ‘pathogenic’ SNVs in ExAC*** | **‘Pathogenic’ missense and nonsense SNVs identified in mutation repositories present in ExAC (number of alleles observed) [number of alleles from TCGA cohort]§** |
| --- | --- | --- | --- | --- | --- | --- | --- |
| **Familial Hypocalciuric Hypercalcemia (FHH)** | *CASR* | 1/20,000-80,000 | 60-70% | ~100% | c. 1/7500 | c.1/3500 | Reported pathogenic/likely pathogenic:  c.32T>C; p.Leu11Ser (1)  c.73C>T; p.Arg25X (2)  c.427G>A; p.Glyy143Arg (1)  c.788C>T; p.Thr263Met (1)  c.1393C>T; p.Arg465Trp (2) [1]  c.1525G>A; p.Gly509Arg (1)  c.1693A>G; p.Ile565Val (1)  c.1972C>T; p.Arg658X (1)  c.2068C>T; p.Arg690Cys (1)  c.2069G>A; p.Arg690His (2)  c.3043G>A; p.Asp1015Asn (4) [1]  c.493-1G>A (splice) (1) |
| **Hyperparathyroidism-Jaw Tumor syndrome (HPT-JT)** | *CDC73* | <1/100,000 | ~100% | 50-70% | c. 1/60,000 | c.1/60,000 | Reported pathogenic/likely pathogenic:  c.815A>G; p.Asn272Ser (1) |
| **Multiple Endocrine Neoplasia type 1 (MEN1)** | *MEN1* | 1/30,000 | 90-100% | 100% | None observed | c. 1/2000-1/20,000 | Reported pathogenic/likely pathogenic:  c.644C>T;p.Thr215Ile (1)  c.958G>T; pAsp320Tyr (1)  c.1354T>C; p.Phe452Leu (1)  Conflicting reports of pathogenicity:  c.113A>T; p.Glu371Asp (10)  c.1609G>T; p.Gly537Cys (2) [2]  c.1633C>T; p.Pro545Ser (11) [1] |
| **Multiple Endocrine Neoplasia type 2 (MEN2)** | *RET* | 1/80,000 | ~100% | 50-100% | c. 1/60,000 | c. 1/1750 | Reported pathogenic/likely pathogenic:  c.1852T>C; p.Cys618Arg (1) [1]  c.1900T>C; pCys634Arg (1)  c.1901G>T; p.Cys634Phe (1)  c.2370G>C; p.Leu790Phe (1)  c.2370G>T; p.Leu790Phe (2)  c.2410G>T; pVal804Leu (1)  c.2410G>A; p.Val804Met (13) [2]  c.2735G>C; p.Arg912Pro (2) [1] |
| **Neurofibromatosis type 1 (NF-1)** | *NF1* | 1/3000 | 100% | ~100% | c. 1/3000 | c.1/3000-1/5000 | Reported pathogenic/likely pathogenic:  c.3314+1G>A (splice) (1)  c.4430+1G>A (splice) (1)  c.574C>T; p.Arg192X (1)  c.1246C>T; p.Arg416X (1)  c.1318C>T; p.Arg440X (1)  c.2041C>T; p.Arg681X (1)  c.2246C>G; p.Ser749X (1)  c.3826C>T; p.Arg1276X (1)  c.4084C>T; p.Arg1362X (1) [1]  c.1885G>A; pGly629Arg (2)  Conflicting Pathogenicity:  c.2339C>A; p.Thr780Lys (1) [1]  c.3467A>G; p.Asn1156Ser (1)  4330A>G; p.Lys1444Glu (1)  5225A>G; p.Asn1742Ser (5) [1] |
| **Von Hippel Lindau (VHL)** | *VHL* | 1/60,000 | 100% | ~100% | c.1/7500 | c.1/1000-1/6000 | Reported pathogenic/likely pathogenic:  c.154G>T; p.Glu52X (1)  c.208G>A; p.Glu70Lys (1)  c.407T>C; p.Phe136Ser (1) [1]  c.499C>T; p.Arg167Trp (1) [1]  c562C>G; p.Leu188Val (2) [2]  Conflicting reports pathogenicity:  c.241C>T; p.Pro81Ser (20) [4]  c.538A>G; p.Ile180Val (1)  c.556G>A; p.Glu186Lys (2) [1]  c.598C>T; p.Arg200Trp (28) [4] |

Sources of disease-associated SNVs: FHH, CASRdb (http://www.casrdb.mcgill.ca/), HGMD (http://www.hgmd.cf.ac.uk/ac/gene.php?gene=CASR) and ClinVar (http://www.ncbi.nlm.nih.gov/clinvar); HPT-JT, reported in Newey *et al*, *Human Mutation* (2010)(1); MEN1 reported in Lemos *et al*, *Human Mutation* (2008)(2), UMD-MEN1 mutation database (<http://www.umd.be/MEN1/>) and ClinVar; MEN2, ARUP Scientific Resource (http://www.arup.utah.edu/database/men2/men2_welcome.php) and ClinVar; NF-1, LOVD (https://grenada.lumc.nl/LOVD2/mendelian_genes/home.php?select_db=NF1) and ClinVar; VHL, HGMD (http://www.hgmd.cf.ac.uk/ac/gene.php?gene=VHL) and ClinVar. (Databases accessed April 2016 – January 2017). It should be noted that the databases employed do not necessarily represent a comprehensive set of pathogenic variants but rather only those that have been submitted/reviewed at the relevant collections.

*Note is made where variants have been reported to have conflicting estimates of pathogenicity (e.g. variant reported as pathogenic/likely pathogenic at one or more sites, but as a VUS at another) as observed for *MEN1*, *NF-1* and *VHL*. In such cases the lower range of estimated prevalence of pathogenic alleles includes these variants, whilst the upper range only includes variants consistently reported as pathogenic/likely pathogenic. Nomenclature according to gene transcript identifiers (which may differ from mutation nomenclature in the literature); *CASR*, ENST00000498619; *CDC73* ENST00000367435; *MEN1,* ENST00000337652; *NF1,* ENST00000358273; *RET*, ENST00000355710; *VHL*, ENST00000256474.

§ To investigate the possibility that the inclusion of n=7601 germline samples from The Cancer genome Atlas (TCGA) cohort (~12.5% of total ExAC population) may result in an overrepresentation of disease-associated variants in hereditary endocrine tumor genes, the number of alleles derived from the TCGA cohort was established for each of the genes. Thus, in addition to the total number of alleles observed in the ExAC population (provided in parentheses), the number of alleles derived from the TCGA cohort is shown in square brackets [ ]. This information is provided for both cancer and non-cancer predisposition genes. No enrichment of alleles is observed from the TCGA cohort with the potential exception of *VHL*, in which 4/6 likely pathogenic alleles occurred in individuals from the TCGA cohort. However, phenotype information on individual variant carriers is not available, such that it is not possible to identify the types of cancer present in *VHL* variant carriers. However, the inclusion of 344 individuals with renal clear cell carcinoma in the TCGA cohort raises the possibility that the inclusion of this cancer type may have resulted in overrepresentation of pathogenic *VHL* alleles in the ExAC population.

Abbreviations; LOF, loss of function

**Supplemental Table 8: Clinically ‘actionable’ variants in hereditary endocrine genes as recommended by ACMG guidelines**

| **Gene** | **Predicted** | **Variant type** | **Number of alleles observed (alleles occuring in TCGA cohort*)** |
| --- | --- | --- | --- |
| ***SDHB*** | p.Gly96Ser | missense | 1(1) |
| ***SDHB*** | p.Gly96Asp | missense | 1(1) |
| ***SDHB*** | p.Arg230Cys | missense | 1 |
| ***SDHB*** | p.Arg242His | missense | 3 |
| ***SDHB*** | p.Arg27Ter | stop gained | 1 |
| ***SDHB*** | p.Arg46Ter | stop gained | 2 |
| ***SDHB*** | p.Arg90Ter | stop gained | 1 |
| ***SDHB*** | p.Arg115Ter | stop gained | 2 |
| ***SDHB*** | c.423+1G>A | splice donor | 1 |
|  |  |  |  |
| ***SDHC*** | p.Arg72Cys | missense | 1 |
| ***SDHC*** | p.Arg133Ter | stop gained | 3 |
|  |  |  |  |
| ***SDHD*** | p.His102Arg | missense | 1 |
|  |  |  |  |
| ***MEN1*** | p.Thr215Ile | missense | 1 |
| ***MEN1*** | pAsp320Tyr | missense | 1 |
| ***MEN1*** | p.Phe452Leu | missense | 1 |
|  |  |  |  |
| ***RET*** | p.Cys618Arg | missense | 1(1) |
| ***RET*** | p.Cys634Arg | missense | 1 |
| ***RET*** | p.Cys634Phe | missense | 1 |
| ***RET*** | p.Leu790Phe | missense | 1 |
| ***RET*** | p.Leu790Phe | missense | 2 |
| ***RET*** | p.Val804Leu | missense | 1 |
| ***RET*** | p.Val804Met | missense | 13(2) |
| ***RET*** | p.Arg912Pro | missense | 2(1) |
|  |  |  |  |
| ***VHL*** | p.Glu52Ter | Stop gained | 1 |
| ***VHL*** | p.Glu70Lys | missense | 1 |
| ***VHL*** | p.Phe136Ser | missense | 1(1) |
| ***VHL*** | p.Arg167Trp | missense | 1(1) |
| ***VHL*** | p.Leu188Val | missense | 2(2) |
|  |  |  |  |
|  |  | **Total** | **49** |

Annotated as reported as per canonical transcript which may differ from nomenclature in relevant databases/literature

Only variants reported in existing databases as pathogenic/likely pathogenic were included (i.e. variants with conflicting reports of pathogenicity were excluded) which may significantly underestimate overall prevalence of reportable mutations as additional LOF alleles are observed for *VHL*, *SDHC* and *SDHD*. Mutations were identified from respective mutation database. For *RET*, *MEN1* and *VHL* these were as reported in footnote for Supplementary Table 7. For *SDHX* genes, mutations were identified from ClinVar and from the curated TCA Cycle Gene Mutation Database (<http://chromium.lovd.nl/LOVD2/SDH/home.php>)

*To investigate the possibility that the inclusion of n=7601 germline samples from the The Cancer Genome Atlas (TCGA) cohort in the ExAC study population, may result in an overrepresentation of disease-associated variants in hereditary tumor genes, we determined the number of alleles derived from the TCGA cohort (shown in parentheses). In total, no statistical significant excess of variants was derived from the TCGA cohort (p>0.1; Fisher’s exact test), although it is notable that 4 of 6 *VHL* variants occurred in individuals from the TCGA cohort, raising the possibility that the inclusion of the TCGA cohort in ExAC might have resulted in an excess of potentially pathogenic VHL variants (See also Supplemental Table 7).

**Supplemental Table 9: Studies of germline *AIP* variants in sporadic pituitary tumors**

| **Study (lead author)** | **Samples (n)*** | **Total *AIP* mutation (n)** | **LOF *AIP* mutations**  **(n)** | **Total Missense *AIP* Mutations§ (n)** | **Other *AIP* missense variants with AF<0.5% reported VUS or benign (n)** | **Notes** |
| --- | --- | --- | --- | --- | --- | --- |
| Tichomirowa *et al* (3) | 163 | 19 | 9 | 8 | 3 (Arg16His (2), Arg128His (1)) | Glu197Glu and Phe269Phe reported as mutations |
| Ferrau *et al* (4) | 203 | 4 | 1 | 3 | 1 (Arg16His) |  |
| Lentiou *et al* (5) | 85 | 0 | 0 | 0 | 0 |  |
| Georgitsu *et al* (6) | 369 | 7 | 6 | 1 | 5 (Arg16His (5)) |  |
| Barlier *et al* (7) | 148 | 1 | 1 | 0 | 0 |  |
| Cazabat *et al* (2007) (8) | 154 | 6 | 5 | 1 | 2 (Arg16His (2)) |  |
| Cazabat *et al* (2012) (9) | 443 | 16 | 7 | 9 | 0 | Arg9Gln and Arg16His reported as missense mutations |
| Cuny *et al* (10) | 127 | 15 | 12 | 3 | 0 |  |
| Preda *et al* (11) | 174 | 2 | 1 | 1 | 1 (Arg16His) |  |
|  |  |  |  |  |  |  |
| **Total** | **1866** | **70** | **42** | **26** | **12** |  |

*includes samples reporting germline variants in association with apparently sporadic pituitary adenomas (i.e. FIPA samples excluded from analysis)

**§** Mutations were annotated in the table as reported in the literature. For example, Arg16His was typically reported as a VUS/benign change but reported as a mutation in Cazabat et al 2012. Abbreviations: AF, allele frequency; LOF, loss of function; VUS, variant unknown significance.

**Supplemental Table 10: Estimate of expected number of individuals with pituitary macroadenomas due to *AIP* mutation in ExAC population**

| **Estimated pituitary macroadenoma prevalence*** | **Expected No. in ExAC Population with macroadenoma^§^** | **Expected No. with macroadenoma due to *AIP* mutation^±^** | **Estimated *AIP* mutation Penetrance^∞^** | **Expected No. in ExAC with ‘at risk’ *AIP* mutation ♯** | **Expected No. in ExAC with LOF *AIP* mutation^$^** | **Expected No. in ExAC with Missense *AIP* mutation** |
| --- | --- | --- | --- | --- | --- | --- |
|  |  |  |  |  |  |  |
| 32/100,000 | 19.3 | 0.77 | 20% | 3.8 | 2.3 | 1.5^⌘^ |
|  |  |  |  |  |  |  |

*Includes macroprolactinoma, non-functioning pituitary macroadenomas and pituitary somatotrophinomas. Prevalence estimates base on Fernandez et al, 2010 in which the prevalence of all pituitary tumor subtypes including microadenomas was estimated at 77.6 cases/100,000, of which non-functioning, prolactin-secreting and GH-secreting macroadenomas represented ~41% giving an estimated prevalence of 32/100,000 in the population.

§ ExAC population size ~60,700

± Assuming 4% *AIP* mutation frequency in pituitary tumor subtypes as reported in the literature

∞ Typical penetrance reported for AIP mutations

♯ Derived by multiplying expected number of individuals with pituitary tumor due to *AIP* mutation by 5 (accounting for the ~20% penetrance of disease)

**^$^** LOF variants reported to represent ~60% of AIP mutations in sporadic pituitary tumors. Notably 2 individuals are observed to harbor *AIP* LOF alleles in the ExAC cohort

^⌘^ Estimated number of individuals in ExAC cohort predicted to have ‘clinically relevant’ missense *AIP* mutation based on existing literature

Abbreviation: LOF, loss of function

**Supplemental Figures**

**
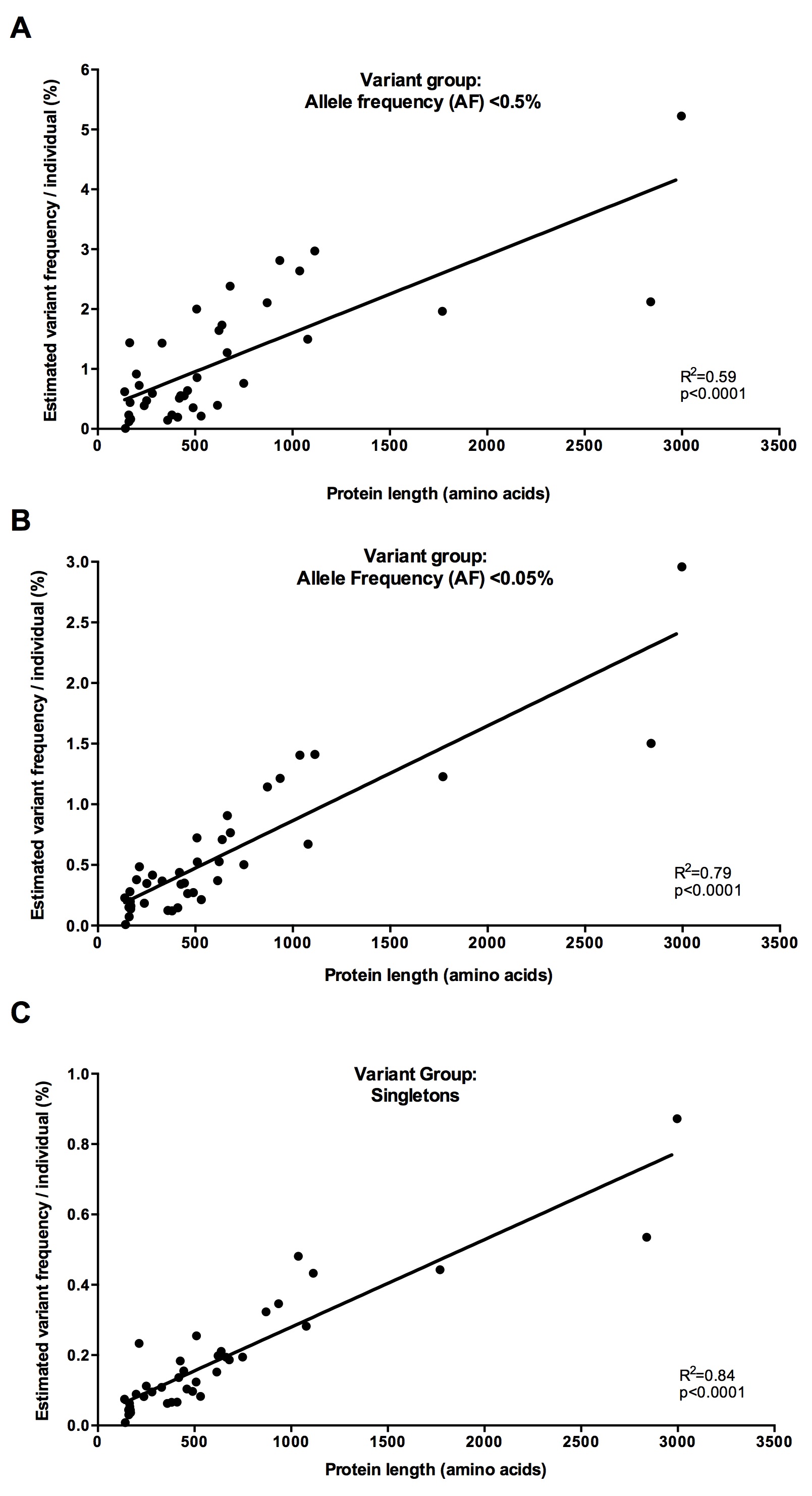
**

**Supplemental Fig 1. Gene-level estimates of cumulative rare SNV frequency correlate with protein size**

For each of the 38 genes under study, cumulative rare non-synonymous (missense/LOF) variant frequencies were established for each of the three categories of SNV (i.e. Allele Frequency (AF) <0.5%, AF <0.05%, and singletons) and expressed as a cohort level prevalence (i.e. percentage of cohort carrying a gene-specific rare missense/LOF SNV in each of the respective variant categories) and plotted against the amino acid length of the respective protein. For each of the three categories of rare SNV, AF <0.5% (**A**), AF <0.05% (**B**) and singletons (**C**) a significant correlation was observed between the gene-levels estimates of cumulative SNV frequency and protein length. The strength of correlation was strongest for singleton variants (r^2^=0.84, p<0.0001), although was evident for both AF <0.05% (r^2^=0.79, p<0.0001) and AF <0.5% (r^2^=0.59, p<0.0001) groups. Analysis performed using linear regression and Pearson’s Correlation


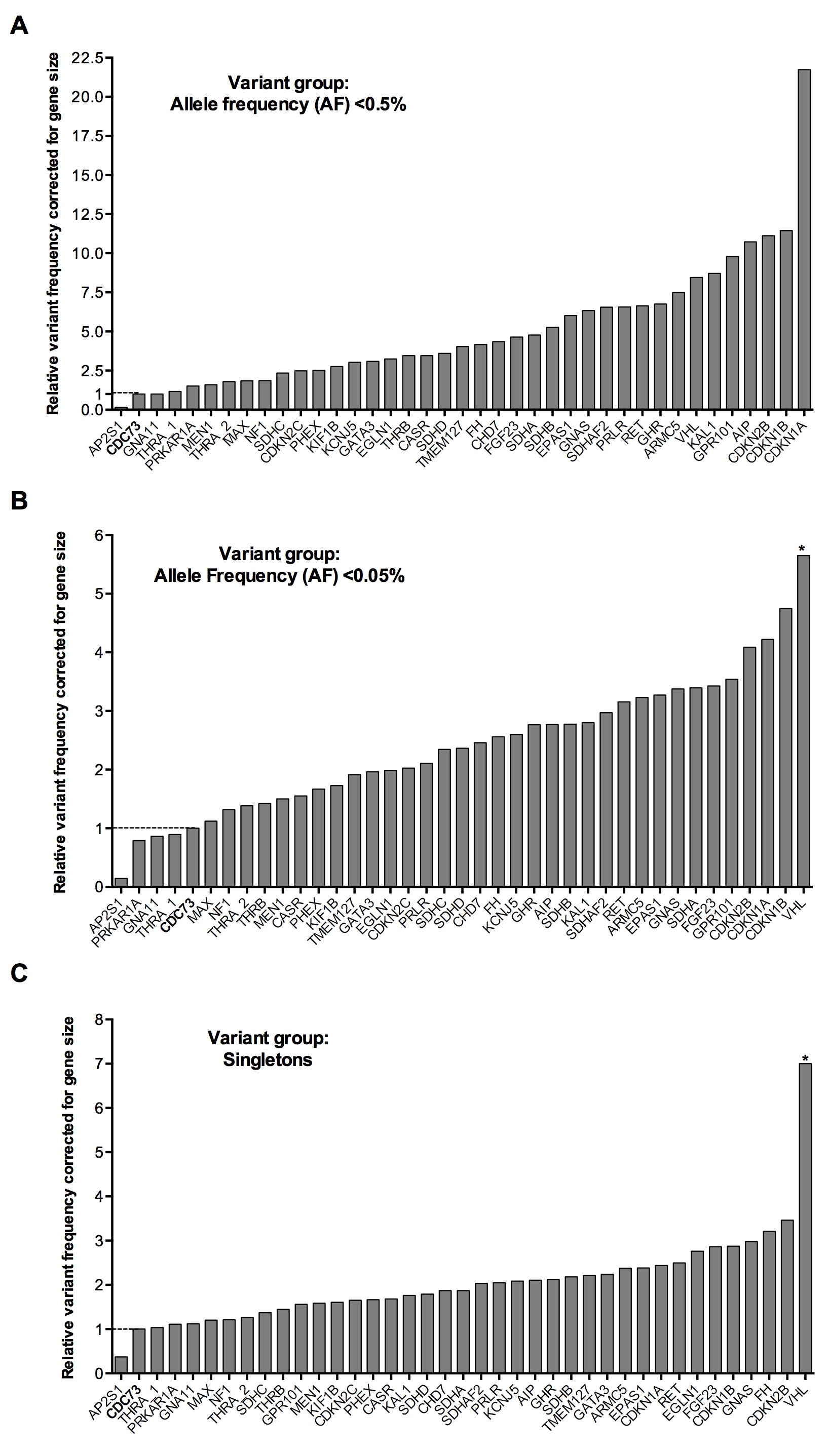


**Supplemental Fig 2. Estimated rare SNV frequencies corrected for protein size**

To correct for gene-size, the cumulative frequencies of each category of rare variant (i.e. AF <0.5%, AF <0.05%, and singletons) were divided by coding-region nucleotide length of the respective gene. To quantify the variability in size-corrected variant frequencies between genes, values were expressed relative to a arbitrarily selected reference gene, *CDC73,* chosen due to observed low size-corrected variant frequencies across each rare SNV group but not representing a significant outlier (e.g. as observed for *AP2S1*)). This analysis demonstrates marked variability between genes for each category of rare variant (i.e. AF <0.5% (**A**), AF <0.05% (**B**), and singleton variants (**C**). Thus, excluding notable outliers (e.g. *AP2S1, CDKN1A, VHL*), the relative size-corrected rare SNV frequency varied ~12-fold between genes in the AF <0.5% group, ~6 fold for the AF< 0.05% group, and 3-fold for singletons. The high singleton rate for *VHL* (marked*) is likely inaccurate due to incomplete sequence data of the *VHL* gene (e.g. missing data and reduced depth of coverage). Thus, singletons occurring in regions with reduced numbers of alleles captured will elevate the apparent singleton SNV frequency.

**
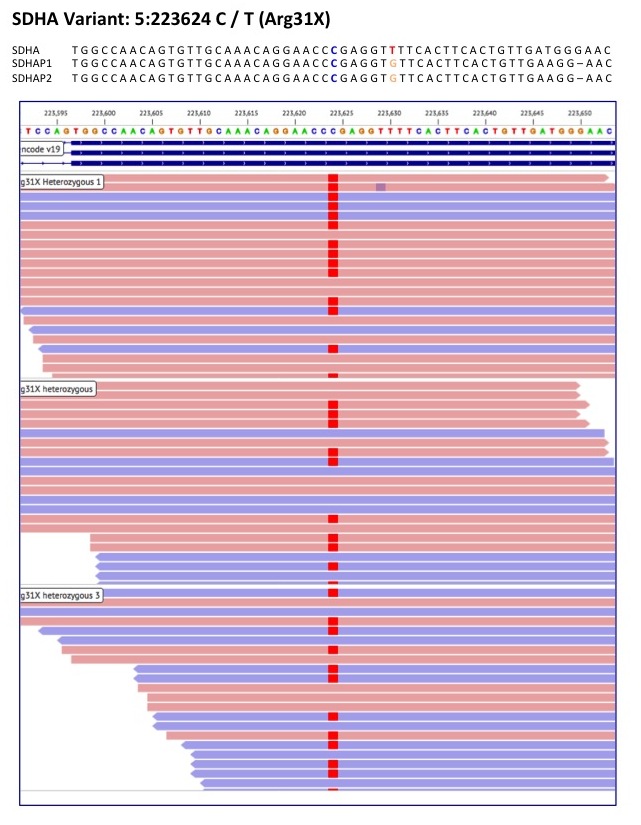
**

**Supplemental Fig. 3. Visual confirmation of SNVs in genes with pseudogenes**

The reliable identification of SNVs occurring in genes with one or more pseudogenes may present a specific challenge. For example, *SDHA* has at least 3 pseudogenes (*SDHAP1*, *SDHAP2*, *SDHAP3*), which share ≥95% sequence homology with a significant proportion of the coding region. The visual inspection of sequence reads obtained from exome sequencing may increase the confidence that individual SNVs are correctly annotated and are present in the gene under study rather than the associated pseudogene(s). Thus, in the example above, it is evident that the C>T nucleotide variant present at position 223624 is present in *SDHA* rather than its associated pseudogenes, as the individual sequence reads harbor the nucleotide T at position 223630 (i.e. 5 nucleotides downstream of the variant), which would be anticipated to be a G nucleotide in the two corresponding pseudogenes.

**Supplemental Materials and Methods**

**ExAC Non-TCGA Analysis**

The ExAC study population includes 7601 germline samples from The Cancer Genome Atlas (TCGA) cohort representing individuals with a diverse range of sporadic cancers (Supplemental Table 1). Notably, endocrine tumors directly relevant to the genes under study are not present in this cohort (e.g. Pheochromocytoma/paraganglioma (PPGL), Medullary Thyroid Cancer (MTC), pituitary tumors). However, the cohort does contain tumor types with potential relevance to genes associated with hereditary endocrine tumor syndromes. For example, it is plausible that a low percentage of individuals with sporadic clear cell renal carcinoma may harbor an underlying germline *VHL* mutations, whilst germline *SDHx* mutations have been reported in some non-PPGL cancers. Similarly, individual hereditary endocrine tumor syndromes have been associated with an increased risk of certain tumor types (e.g. Hyperparathyroidism-Jaw Tumor (HPT-JT) syndrome and uterine lesions). Thus, to investigate the possibility that the inclusion of the TCGA cohort in the ExAC population may have resulted in an overrepresentation of rare SNVs in hereditary endocrine tumor genes, a separate analysis was performed on the ExAC cohort with the TCGA samples removed (non-TCGA cohort) (n=53,105). This dataset (ExAC_nonTCGA.r0.3.1) was downloaded at ftp://ftp.broadinstitute.org/pub/ExAC_release/release0.3.1/subsets/. Thus, all rare SNVs (AF <0.5%, AF <0.05% and singletons) were identified in 12 genes associated with hereditary endocrine tumors (Supplemental Table 4). Cumulative rare variant frequencies were estimated for each gene by accruing individual SNV frequencies and expressed as a ‘Number-Needed-to-Sequence’ (‘NNS’) and compared to those for the complete ExAC cohort (Supplemental Table 4). In addition, the non-TCGA cohort was used to identify which of the apparently disease-causing SNVs identified in the ExAC population arose in TCGA samples (Supplemental Tables 7 and 8).

**Identification of Small Insertions and Deletions (Indels) in ExAC cohort**

Although whole exome sequencing has the potential to identify small insertions or deletions (indels), a number of technical challenges result in a reduced reliability for detection. For the ExAC population the sensitivity and false discovery rate (FDR) for indels is reported as 95% and 2.2%, respectively (relative to ‘platinum standard’ genomes sequenced using multiple technologies) (12). This compares with a sensitivity and FDR for SNVs of 99.8% and 0.056%. Thus, due to the potential reduced reliability of detection, we excluded indels from the main analysis. However, accepting these potential limitations, we performed a separate analysis to quantify the frequency of indels predicted to result in a loss of gene function (i.e. frameshift) in each of the 38 genes (Supplemental Table 6). The frequency of LOF indels was estimated using the same methods as for SNVs.

**Constraint Metrics**

Missense ‘Z-score’) and LOF ‘probability of loss-of function intolerance’ (‘pLI’) constraint metrics for individual genes were obtained directly from ExAC browser. Extensive descriptions of the derivation of the metrics is provided elsewhere (12). In brief, for missense SNVs, the Z-score represents the predicted deviation of observed SNVs from an expected number deduced by evaluating the probability of each trinucleotide repeat mutating to all other possible trinucleotides(12). A positive Z score is suggestive of increased constraint (i.e. intolerant of variation) whilst a negative Z scores indicates a gene with more missense variants than expected (i.e. tolerant of variation). A Z-score of >3.09 defines significant intolerance (P<0.001), but requires a minimum of 10 expected variants. For evaluation of loss-of-function (LOF) tolerability, the observed and expected variant counts were used to generate a ‘probability of being loss of function intolerant’ (pLI) metric. This metric assumes genes fall into one of 3 groups: null (i.e. LOF completely tolerated); recessive (i.e. heterozygous LOF tolerated, but homozygote LOF not); and haploinsufficient (i.e. heterozygous LOF not tolerated). The closer pLI is to 1, the more likely the gene is intolerant to LOF, with pLI scores >0.9 considered indicative of extreme LOF intolerance and a haploinsufficiency function. Across the ExAC cohort the pLI distribution is reported to be approximately bi-modal with most genes considered either tolerant (pLI<0.1) or severely intolerant (pLI>0.9). In prior analyses pLI, outperformed other metrics of LOF intolerance (12). For both missense and LOF SNVs, the number of predicted and observed variants reflects unique variants (i.e. does not take into account multiple observations of the same allele). For LOF analysis, only SNVs resulting in nonsense amino acid changes of those directly affecting splice donor or splice acceptor sites are included (i.e. exclusion of indels).

**Predictive SNV Computational Tools**

The utility of 3 currently employed computational tools used to predict variant pathogenicity were evaluated:

SIFT (http://sift.jcvi.org); Polyphen2 (<http://genetics.bwh.harvard.edu/pph2>); and the Combined Annotation Dependent Depletion (CADD) (<http://cadd.gs.washington.edu/>). Scores for each of these tools for all missense SNVs identified in ExAC were available for download at: <http://cadd.gs.washington.edu/> (accessed April 2016). In this study, we selected 12 genes in which missense SNVs have been reported in association with highly penetrant (*MEN1*) or moderately penetrant (e.g. *AIP*, *SDHB*) disease. Scores for each of SIFT, Polyphen2 and CADD were evaluated for all missense SNVs with Allele Frequency (AF) <0.5% in the 12 selected genes. Scoring for each of the tools is described elsewhere (i.e. see respective homepage) but in summary: For SIFT, variants are classified as ‘damaging/deleterious’ if SIFT ≤0.05, and ‘tolerated’ if ≥0.05; for Polyphen2, variants are classified as ‘probably damaging’, ‘possibly damaging’ or ‘benign’; and for CADD, scaled scores with a cut-off ≥20 were employed (representing the top 1% of deleteriousness(13)). We classified variants as ‘deleterious’ if they met all of the following criteria: AF<0.5%, SIFT<0.05, Polyphen2 category ‘probably damaging’ and scaled CADD score >20. We classified variants as ‘possibly deleterious’ if they met the following criteria: AF<0.5% and either SIFT ≤0.05 or Polyphen2 grading of ‘probably damaging’ or ‘possibly damaging’.

**Prevalence of disease-associated variants in ExAC cohort**

The ExAC cohort was evaluated for the presence of missense and LOF SNVs previously reported to be disease-causing in six penetrant monogenic conditions (Familial Hypocalciuric Hypercalcaemia (FHH), Multiple Endocrine Neoplasia type 1 (MEN1), Multiple Endocrine Neoplasia type 2 (MEN2), Hyperthyroidism-Jaw Tumor Syndrome (HPT-JT), Neurofibromatosis Type 1 (NF-1), von Hippel Lindau (VHL)). Disease-associated mutations were identified from publicly available mutation repositories, disease-specific databases or from mutation-collections reported in the literature. Sources of disease-associated SNVs for the respective conditions were: FHH, CASRdb (http://www.casrdb.mcgill.ca/), HGMD (http://www.hgmd.cf.ac.uk/ac) and ClinVar (http://www.ncbi.nlm.nih.gov/clinvar); HPT-JT, reported in Newey *et al*, 2010(1); MEN1 reported in ClinVar, UMD-MEN1 mutation database (<http://www.umd.be/MEN1/>) and Lemos et al, 2008(2); MEN2, ARUP Scientific Resource (http://www.arup.utah.edu/database/men2/men2_welcome.php) and ClinVar; NF-1, LOVD (https://grenada.lumc.nl/LOVD2/mendelian_genes/home.php) and ClinVar; and VHL, HGMD and ClinVar.. The presence of each mutation was evaluated in the ExAC cohort. In each case nomenclature for the canonical transcript was used which may differ from the variant description in the respective disease-specific database. Conflicting reports of variant pathogenicity were also noted (see Supplemental Table 7). Additionally, potentially actionable *SDHX* (i.e. *SDHA*, *SDHB, SDHC, SDHD, SDHAF2*) variants were identified from ClinVar and LOVD TCA cycle mutation database (<http://chromium.lovd.nl/lovd_sdh/variants.php?action=search_unique>). Only variants reported as disease causing in existing databases/mutation collections were included in the analysis.

**Supplemental References**

1. Newey PJ, Bowl MR, Cranston T, Thakker RV. Cell division cycle protein 73 homolog (CDC73) mutations in the hyperparathyroidism-jaw tumor syndrome (HPT-JT) and parathyroid tumors. Hum Mutat. 2010; 31:295-307

2. Lemos MC, Thakker RV. Multiple endocrine neoplasia type 1 (MEN1): analysis of 1336 mutations reported in the first decade following identification of the gene. Hum Mutat. 2008; 29:22-32

3. Tichomirowa MA, Barlier A, Daly AF, Jaffrain-Rea ML, Ronchi C, Yaneva M, Urban JD, Petrossians P, Elenkova A, Tabarin A, Desailloud R, Maiter D, Schurmeyer T, Cozzi R, Theodoropoulou M, Sievers C, Bernabeu I, Naves LA, Chabre O, Montanana CF, Hana V, Halaby G, Delemer B, Aizpun JI, Sonnet E, Longas AF, Hagelstein MT, Caron P, Stalla GK, Bours V, Zacharieva S, Spada A, Brue T, Beckers A. High prevalence of AIP gene mutations following focused screening in young patients with sporadic pituitary macroadenomas. Eur J Endocrinol. 2011; 165:509-515

4. Ferrau F, Romeo PD, Puglisi S, Ragonese M, Torre ML, Scaroni C, Occhi G, De Menis E, Arnaldi G, Trimarchi F, Cannavo S. Analysis of GPR101 and AIP genes mutations in acromegaly: a multicentric study. Endocrine. 2016;

5. Leontiou CA, Gueorguiev M, van der Spuy J, Quinton R, Lolli F, Hassan S, Chahal HS, Igreja SC, Jordan S, Rowe J, Stolbrink M, Christian HC, Wray J, Bishop-Bailey D, Berney DM, Wass JA, Popovic V, Ribeiro-Oliveira A, Jr., Gadelha MR, Monson JP, Akker SA, Davis JR, Clayton RN, Yoshimoto K, Iwata T, Matsuno A, Eguchi K, Musat M, Flanagan D, Peters G, Bolger GB, Chapple JP, Frohman LA, Grossman AB, Korbonits M. The role of the aryl hydrocarbon receptor-interacting protein gene in familial and sporadic pituitary adenomas. J Clin Endocrinol Metab. 2008; 93:2390-2401

6. Georgitsi M, Raitila A, Karhu A, Tuppurainen K, Makinen MJ, Vierimaa O, Paschke R, Saeger W, van der Luijt RB, Sane T, Robledo M, De Menis E, Weil RJ, Wasik A, Zielinski G, Lucewicz O, Lubinski J, Launonen V, Vahteristo P, Aaltonen LA. Molecular diagnosis of pituitary adenoma predisposition caused by aryl hydrocarbon receptor-interacting protein gene mutations. Proc Natl Acad Sci U S A. 2007; 104:4101-4105

7. Barlier A, Vanbellinghen JF, Daly AF, Silvy M, Jaffrain-Rea ML, Trouillas J, Tamagno G, Cazabat L, Bours V, Brue T, Enjalbert A, Beckers A. Mutations in the aryl hydrocarbon receptor interacting protein gene are not highly prevalent among subjects with sporadic pituitary adenomas. J Clin Endocrinol Metab. 2007; 92:1952-1955

8. Cazabat L, Libe R, Perlemoine K, Rene-Corail F, Burnichon N, Gimenez-Roqueplo AP, Dupasquier-Fediaevsky L, Bertagna X, Clauser E, Chanson P, Bertherat J, Raffin-Sanson ML. Germline inactivating mutations of the aryl hydrocarbon receptor-interacting protein gene in a large cohort of sporadic acromegaly: mutations are found in a subset of young patients with macroadenomas. Eur J Endocrinol. 2007; 157:1-8

9. Cazabat L, Bouligand J, Salenave S, Bernier M, Gaillard S, Parker F, Young J, Guiochon-Mantel A, Chanson P. Germline AIP mutations in apparently sporadic pituitary adenomas: prevalence in a prospective single-center cohort of 443 patients. J Clin Endocrinol Metab. 2012; 97:E663-670

10. Cuny T, Pertuit M, Sahnoun-Fathallah M, Daly A, Occhi G, Odou MF, Tabarin A, Nunes ML, Delemer B, Rohmer V, Desailloud R, Kerlan V, Chabre O, Sadoul JL, Cogne M, Caron P, Cortet-Rudelli C, Lienhardt A, Raingeard I, Guedj AM, Brue T, Beckers A, Weryha G, Enjalbert A, Barlier A. Genetic analysis in young patients with sporadic pituitary macroadenomas: besides AIP don't forget MEN1 genetic analysis. Eur J Endocrinol. 2013; 168:533-541

11. Preda V, Korbonits M, Cudlip S, Karavitaki N, Grossman AB. Low rate of germline AIP mutations in patients with apparently sporadic pituitary adenomas before the age of 40: a single-centre adult cohort. Eur J Endocrinol. 2014; 171:659-666

12. Lek M, Karczewski KJ, Minikel EV, Samocha KE, Banks E, Fennell T, O'Donnell-Luria AH, Ware JS, Hill AJ, Cummings BB, Tukiainen T, Birnbaum DP, Kosmicki JA, Duncan LE, Estrada K, Zhao F, Zou J, Pierce-Hoffman E, Berghout J, Cooper DN, Deflaux N, DePristo M, Do R, Flannick J, Fromer M, Gauthier L, Goldstein J, Gupta N, Howrigan D, Kiezun A, Kurki MI, Moonshine AL, Natarajan P, Orozco L, Peloso GM, Poplin R, Rivas MA, Ruano-Rubio V, Rose SA, Ruderfer DM, Shakir K, Stenson PD, Stevens C, Thomas BP, Tiao G, Tusie-Luna MT, Weisburd B, Won HH, Yu D, Altshuler DM, Ardissino D, Boehnke M, Danesh J, Donnelly S, Elosua R, Florez JC, Gabriel SB, Getz G, Glatt SJ, Hultman CM, Kathiresan S, Laakso M, McCarroll S, McCarthy MI, McGovern D, McPherson R, Neale BM, Palotie A, Purcell SM, Saleheen D, Scharf JM, Sklar P, Sullivan PF, Tuomilehto J, Tsuang MT, Watkins HC, Wilson JG, Daly MJ, MacArthur DG, Exome Aggregation C. Analysis of protein-coding genetic variation in 60,706 humans. Nature. 2016; 536:285-291

13. Kircher M, Witten DM, Jain P, O'Roak BJ, Cooper GM, Shendure J. A general framework for estimating the relative pathogenicity of human genetic variants. Nat Genet. 2014; 46:310-315
